# Strain-induced skeletal rearrangement of a polycyclic aromatic hydrocarbon on a copper surface

**DOI:** 10.1038/ncomms16089

**Published:** 2017-07-20

**Authors:** Akitoshi Shiotari, Takahiro Nakae, Kota Iwata, Shigeki Mori, Tetsuo Okujima, Hidemitsu Uno, Hiroshi Sakaguchi, Yoshiaki Sugimoto

**Affiliations:** 1 Department of Advanced Materials Science, The University of Tokyo, 277-8561 Kashiwa, Japan; 2 Institute of Advanced Energy, Kyoto University, 611-0011 Uji, Japan; 3 Graduate School of Engineering, Osaka University, 565-0871 Suita, Japan; 4 Advanced Research Support Center, Ehime University, 790-8577 Matsuyama, Japan; 5 Graduate School of Science and Engineering, Ehime University, 790-8577 Matsuyama, Japan

## Abstract

Controlling the structural deformation of organic molecules can drive unique reactions that cannot be induced only by thermal, optical or electrochemical procedures. However, in conventional organic synthesis, including mechanochemical procedures, it is difficult to control skeletal rearrangement in polycyclic aromatic hydrocarbons (PAHs). Here, we demonstrate a reaction scheme for the skeletal rearrangement of PAHs on a metal surface using high-resolution noncontact atomic force microscopy. By a combination of organic synthesis and on-surface cyclodehydrogenation, we produce a well-designed PAH—diazuleno[1,2,3-*cd*:1′,2′,3′-*fg*]pyrene—adsorbed flatly onto Cu(001), in which two azuleno moieties are highly strained by their mutual proximity. This local strain drives the rearrangement of one of the azuleno moieties into a fulvaleno moiety, which has never been reported so far. Our proposed thermally driven, strain-induced synthesis on surfaces will pave the way for the production of a new class of nanocarbon materials that conventional synthetic techniques cannot attain.

## Abstract (web summary)

Mechanical strains can induce chemical transformations otherwise inaccessible by conventional stimuli. Here, the authors show the unusual strain-induced transformation of a polycyclic aromatic hydrocarbon on a metal surface by means of atomic force microscopy.

Structural strains in organic molecules play a huge role in determining molecular stability. Steric repulsions between component atoms in a hydrocarbon molecule cause the distortion of orbital geometries (such as *sp*^3^ and *sp*^2^ hybridizations) of carbon atoms, giving rise to characteristic reactivity in order to release the strain energy. For instance, the Baeyer strain^1^, an angle strain of *sp*^3^-hydrocarbon rings, is responsible for the ring-opening reactivity of bicyclic compounds^2^. A structural strain can also be applied to molecules by external stimuli; organic synthesis promoted by stress added to reactants is categorized as mechanochemistry^3^,^4^,^5^. Mechanical stimuli, such as high-pressure conditions and ultrasound exposure, can modify molecular geometries to yield specific products that cannot be induced by heat, light or electric fields^6^.

Strain-induced rearrangements of aromatic carbon rings have been observed in graphene structures. As a typical example, a mechanical stress in graphene triggers a Stone–Wales rearrangement^7^,^8^, a typical reaction of carbon allotropes that yields a defect composed of two heptagonal and two pentagonal rings fused together. Such defects play an important role in the formation of curved graphene and fullerenes^8^,^9^,^10^,^11^,^12^. Therefore, it is crucial to control the rearrangements of *sp*^2^-carbon skeletal structures in order to synthesize nanocarbon materials^13^,^14^,^15^. For macromolecules such as graphene, intramolecular rearrangements feasibly occur because such local reactions do not strictly affect the total energy of the huge systems. For small polycyclic aromatic hydrocarbons (PAHs), on the other hand, it has been difficult to induce skeletal rearrangements in conventional organic synthesis. The preceding mechanochemical procedures for aromatic compounds cannot induce unimolecular reactions but can induce *sp*^3^-hybridized polymerizations^16^,^17^ because of the enormous increase in intermolecular interactions. Other synthetic methods under severe conditions, such as flash vacuum pyrolysis^13^,^18^, are required to undermine the aromaticity in the transition states and to obtain intramolecularly rearranged PAHs. Therefore, the exploration of PAH reaction schemes will be a significant step towards understanding the mechanisms underlying the creation and repair of defects in carbon allotropes, and towards designing and fabricating further functional nanocarbon materials.

It is well known that the adsorption of organic molecules onto metal surfaces modifies the molecular geometries and electronic states^19^,^20^. Adsorbate–substrate interactions can promote characteristic thermal reactions (namely, on-surface synthesis^21^,^22^) occurring in neither the crystal nor the solution, which can yield well-defined products such as graphene nanoribbons^23^,^24^,^25^,^26^ and other nanocarbons^27^,^28^,^29^,^30^,^31^. Therefore, we conceive that the geometric structure of a PAH can be distorted by adsorption onto a metal surface, so that the molecule may store strain energy to facilitate a catalytic reaction at the surface. Although some previous studies have implied that the structural distortion of precursors on surfaces is a key factor for on-surface reactions^24^,^26^,^28^,^31^, the correlation between the structural strain and reactivity has not been focused on in the previous studies and remains less well understood. Besides, the binding of a target molecule to a flat surface provides an overwhelming advantage in the characterization of individual molecules on the surface with scanning tunnelling microscopy (STM) and atomic force microscopy (AFM). In particular, noncontact AFM with a functionalized tip (that is, a probe attaching an atom or a molecule to its apex) can visualize chemical bonding structures of adosorbates^32^,^33^,^34^,^35^,^36^,^37^, because it can sensitively detect the repulsive forces between atoms in the adsorbate and the sharpened tip apex^38^,^39^. By using this imaging technique, reactants, intermediates and products of various on-surface reactions have successfully been identified^31^,^40^,^41^,^42^,^43^,^44^,^45^,^46^. We focus on the on-surface reactivity of azulene-derivative PAHs. Azulene (C_10_H_8_), a typical nonbenzenoid aromatic compound composed of seven- and five-membered rings, can be thermally rearranged into its structural isomer of naphthalene^47^,^48^. Such rearrangement of an azulene-type skeletal structure in PAHs is strongly related to the Stone–Wales defects mentioned above^7^,^8^,^49^,^50^. Despite the importance of understanding the reactivity, studies on azulene and its derivatives adsorbed onto surfaces have been scarce^51^,^52^.

Here we show thermally driven intramolecular rearrangement with the assistance of a structural strain of *sp*^2^-aromatic rings. We utilize a metal surface as a tight-binding field to generate a structural strain in the PAH due to the deformation of the molecular structure, a reaction field to catalytically activate intermolecular rearrangement of the PAH, and an observation field to identify the individual reaction products. We newly synthesize geometrically isomeric PAHs with two azuleno moieties. We observe these molecules adsorbed onto a metal surface with STM and noncontact AFM, and compared the difference in reactivity between the PAHs. We verify that the intramolecular structural strain drives the catalytic reaction to rearrange the azuleno moiety.

## Results

### Adsorption of intrinsically distorted PAHs onto Cu(001)

**Figure 1:**
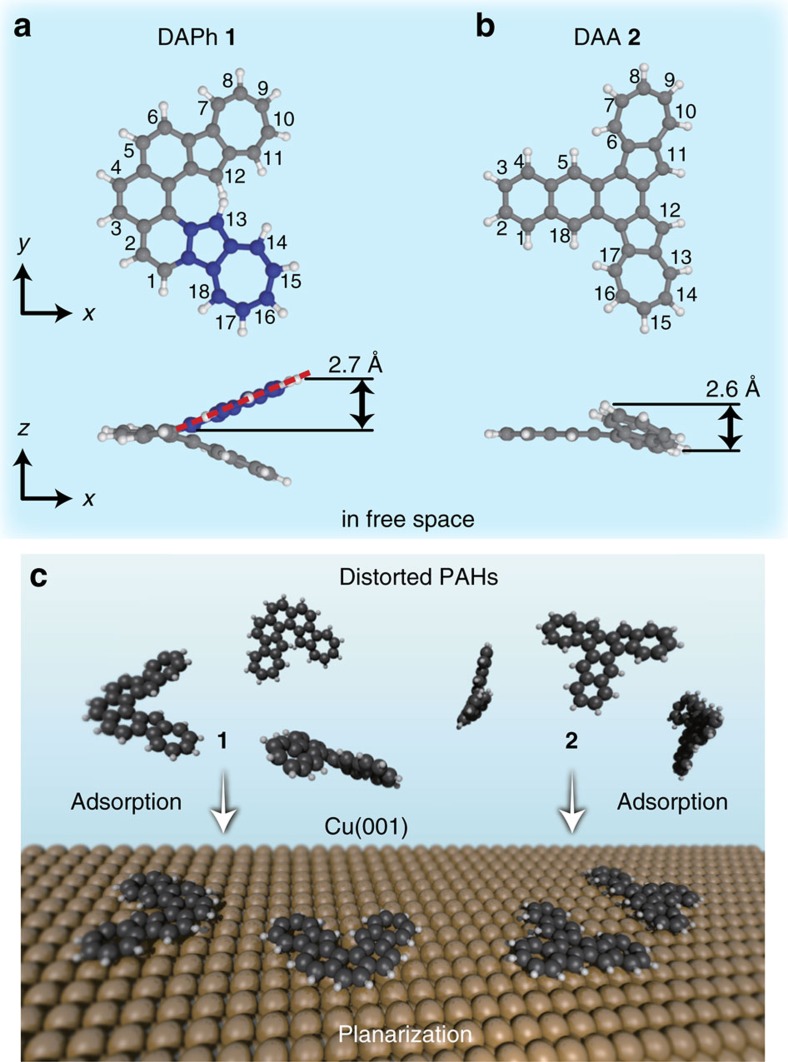
Molecular structures of synthesized azulene-derivative PAHs. (**a**,**b**) Calculated structures of diazuleno[1,2-*c*:2′,1′-*g*]phenanthrene (DAPh) **1** and diazuleno[1,2-*a*:2′,1′-*c*]anthracene (DAA) **2**, respectively, in free space. Numbers at carbon atoms were assigned according to the IUPAC nomenclature. Blue spheres in **a** represent C atoms of one of the azuleno moieties in DAPh **1**. (**c**) Scheme of the adsorption of **1** and **2** onto a Cu(001) surface to modify the molecular geometries.

We synthesized two kinds of PAHs: diazuleno[1,2-*c*:2′,1′-*g*]phenanthrene (DAPh) **1** (Fig. 1a) and diazuleno[1,2-*a*:2′,1′-*c*]anthracene (DAA) **2** (Fig. 1b). The former has a highly twisted geometry in free space because of the steric repulsion between two H atoms bonded to the 12- and 13-positioned C atoms in the azuleno moieties (*C*_2_ point group; Fig. 1a). The latter is a structural isomer of DAPh with a different steric structure: a bent geometry in free space (*C*_s_ point group) because of the H–H repulsions between the 5 and 6 positions and between the 17 and 18 positions. We note that this molecule forms a twisted geometry in the crystal phase (*C*_2_ point group; see Supplementary Data 2). Our calculations of DAA in free space show that the bent configuration is 113 meV more stable than the twisted one. Here we define the molecular height as the distance between the highest- and lowest-*z* atoms when the phenanthrene/anthracene part of the molecule is placed in an *x*–*y* plane. The height of the free DAPh is calculated to be 5.4 Å, indicating that each azuleno moiety is distorted with a height of 2.7 Å (Fig. 1a). Because the free DAA has a molecular height of 2.6 Å (Fig. 1b), the azuleno moieties in both DAPh and DAA molecules are distorted in a similar manner. We target the intrinsically distorted molecules as reactants, because adsorption of the molecules onto a surface is potentially capable of modifying the geometry.

**Figure 2:**
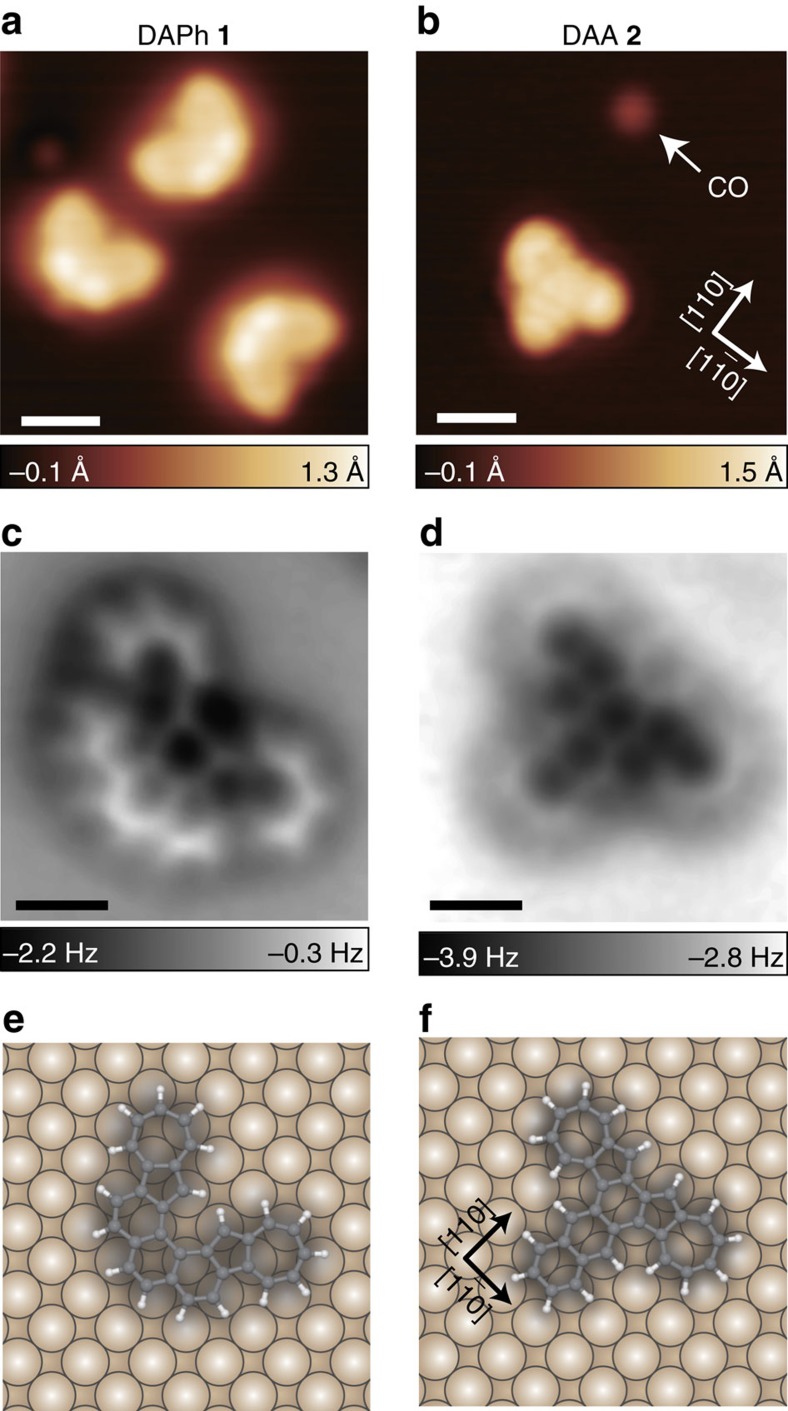
AFM images and adsorption structures of DAPh and DAA on Cu(001). (**a**,**b**) Typical STM images of DAPh **1** and DAA **2**, respectively, on Cu(001) at 4.8 K with CO-terminal tips. (**c**,**d**) AFM images of **1** and **2**, respectively. (**e**,**f**) Schematics of the adsorption structures of **1** and **2**, respectively. The nonplanar PAHs are expected to be flattened on the surface. The adsorption sites were determined experimentally (see Supplementary Fig. 2). The image in **a** was obtained with the sample bias *V*=−30 mV and the tunnelling current *I*=10 pA. The image in **b** was obtained with *V*=30 mV and *I*=20 pA. The image in **c**,**d** was acquired at the tip height corresponding to *V*=50 mV and *I*=200 (20) pA over a bare surface. Scale bars, 10 Å (**a**,**b**); 5 Å (**c**,**d**).

Each isomer we synthesized was adsorbed to a Cu(001) surface (Fig. 1c) and the molecular geometries on the surface were elucidated by using STM/AFM. Figure 2a,b shows topographic STM images of DAPh **1** and DAA **2**, respectively, on the surface at 4.8 K. We used CO-terminal tips^32^ in order to achieve higher spatial resolution in the STM and AFM images (see Supplementary Note 1 and Supplementary Fig. 1). With STM, DAPh (DAA) is imaged as a symmetric V- (T-) shaped protrusion. Figure 2c,d shows AFM images of DAPh and DAA, respectively. In both AFM images, all covalent bonds of the molecule are visualized due to the detection of the repulsive forces between atoms in the adsorbate and CO attached to the tip apex. C–C bonds at the molecular edges are seemingly emphasized because the long-range attractive forces that appear as blurred depressions in the images contribute predominantly at the molecular centre^32^. It is well known that a frequency-shift (Δ*f*) contrast depends strictly on the atom height with respect to the surface; if a molecule is not flat but twisted or bent, the Δ*f* signals in the sterically protruded region in the molecule must be much larger (that is, more repulsive against the probe tip)^31^,^41^,^46^,^53^,^54^,^55^. In the images of DAPh and DAA, on the other hand, all covalent bonds within the molecules are clearly resolved, suggesting that both of the intrinsically distorted PAHs adsorbed flatly onto the surface.

The adsorption sites of these molecules were experimentally determined by STM images at a higher tunnelling current where the molecule and surface Cu atoms are observed simultaneously (Supplementary Note 1 and Supplementary Fig. 2). The adsorption sites of DAPh and DAA are shown in Fig. 2e,f, respectively. Both adsorption structures belong to the *C*_s_ point group with a symmetry axis along the <110> direction. This supports the conclusion that these molecules are flattened on the surface. The modification of the steric structures suggests that the interaction between the molecule and the substrate was strong enough to overcome the geometric distortion of the flattened molecules. According to the adsorption energies of PAHs on Cu surfaces in the literature^20^, the adsorption energies of DAPh and DAA on Cu(001) are estimated to be roughly 4 eV. The flattening energies (that is, the energy between the optimized structure and the geometry that has been totally flattened in free space) of the molecules are calculated to be much lower than the estimated adsorption energies (Table 1). The interaction between all of the component atoms in the flattened molecules and the substrate stabilizes the systems.

### On-surface reactivity of DAPh and DAA

**Figure 3:**
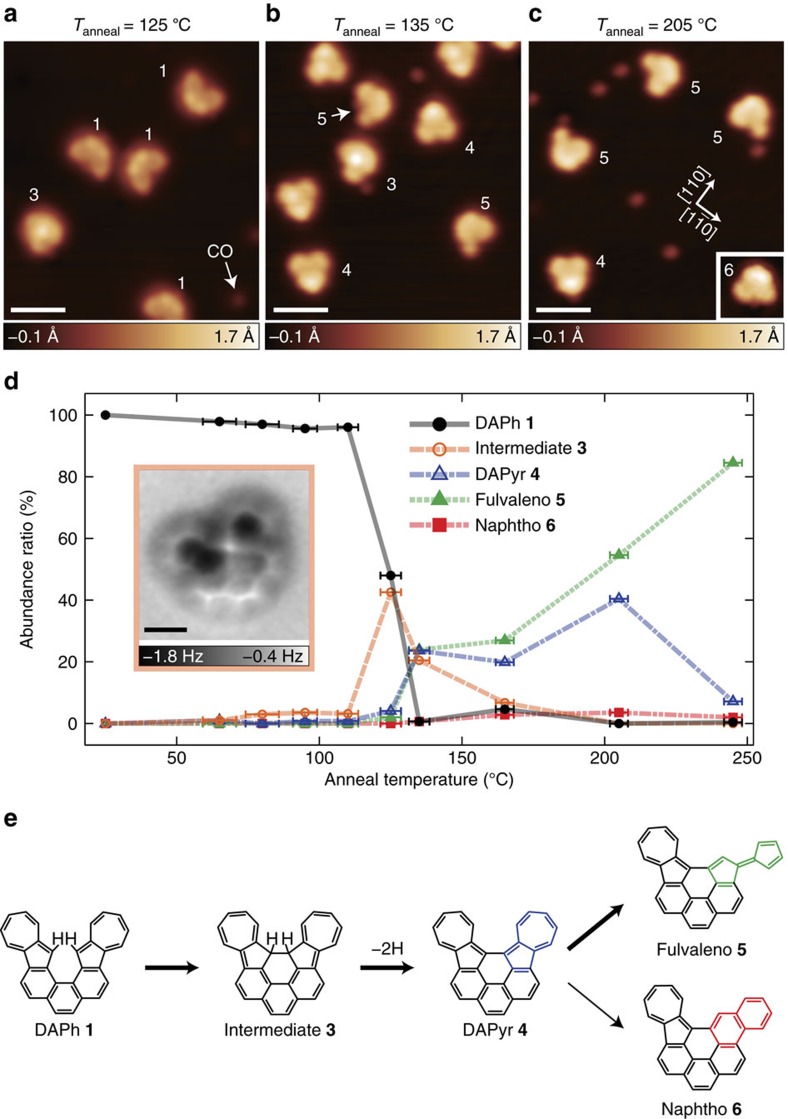
Thermal reaction of DAPh on Cu(001). (**a**–**c**) Typical STM images of DAPh/Cu(001) at 4.8 K with CO terminal tips after annealing for 10 min at 125, 135 and 205 °C, respectively. The inset of **c** shows an STM image of a minor product **6**. (**d**) Annealing-temperature dependence of the abundance ratio of the molecular species on the surface. More than 100 molecules were counted at each annealing temperature. The error bar represents the standard deviation from the set-point temperature. The inset of **d** shows an AFM image of intermediate **3**. (**e**) Reaction of DAPh **1** on Cu(001) to yield a fulvaleno-rearranged product **5** and a naphtho-rearranged product **6** via intermediates **3** and **4**. The images were obtained with *V*=−30 mV and *I*=10 pA for **a**, with *V*=50 mV and *I*=20 pA for **b** and the inset of **d**, and with *V*=50 mV and *I*=200 pA for **c**. The image in the inset of **d** was acquired at the tip height corresponding to *V*=50 mV and *I*=20 pA over a bare surface. Scale bars, 20 Å (**a**–**c**); 5 Å (the inset of **d**).

Next we focus on the thermal reactivity of DAPh and DAA molecules on Cu(001). A sample was annealed and kept at a desired temperature *T*_anneal_ for 10 min, followed by STM/AFM observation at 4.8 K. After the measurements, the sample was cleaned and exposed to the molecular vapour at room temperature again to prepare a sample for another *T*_anneal_. The coverage of the molecules varied between 1–7 × 10^12^ cm^−2^ during the preparation-and-annealing cycles, but no coverage dependence was observed. For DAA **2**, almost none of the adsorbates on Cu(001) reacted and no specific product was observed (see Supplementary Note 2 and Supplementary Fig. 3) even after annealing at *T*_anneal_=165–275 °C. On the other hand, several kinds of products were observed in the post-annealed sample of DAPh/Cu(001). As shown in Fig. 3d, the production yields depended on *T*_anneal_. The pristine DAPh molecules **1** (Fig. 2a) were observed predominantly below *T*_anneal_=110 °C, whereas the yield of another species, **3**, increases at *T*_anneal_=125 °C (Fig. 3a). As shown in Fig. 3a, **3** is clearly characterized by its STM image having a round protrusion near the molecular centre. The chemical assignment of the species is discussed below. Above *T*_anneal_≈130 °C, the yield of **3** decreases gradually with an increase in the abundance ratios of other images, namely, products **4** and **5** (Fig. 3d). With STM, **4** is imaged as a symmetric V-shaped protrusion oriented to the <100> direction, whereas **5** is observed as an asymmetric V-shaped protrusion (Fig. 3b). Figure 3c shows an STM image of the sample at *T*_anneal_=205 °C, where **3** was not observed but **4** and **5** existed predominantly. Above *T*_anneal_=165 °C, we also observed a very minor species **6** (inset of Fig. 3c), with abundance ratios of not more than 3%. **5** became predominant at *T*_anneal_=245 °C, but finally, no molecule was observed at *T*_anneal_=300 °C because all molecules were desorbed from the surface. Figure 3d shows the annealing-temperature dependence of the abundance ratio for **1**, **3**–**6**. Above 125 °C, the sum of the ratios for the five species is not equal to 100% because several kinds of other species (minor products, intermediates and impurities) were also observed (see Supplementary Note 3 and Supplementary Fig. 4). According to the abundance ratios in Fig. 3d, we assume that the thermal reaction pathway of DAPh on the surface is **1**→**3**→**4**→**5**/**6**. Note that this is a tentative assignment and that multiple pathways, for example, **4**→**6**→**5**, might be possible.

**Figure 4:**
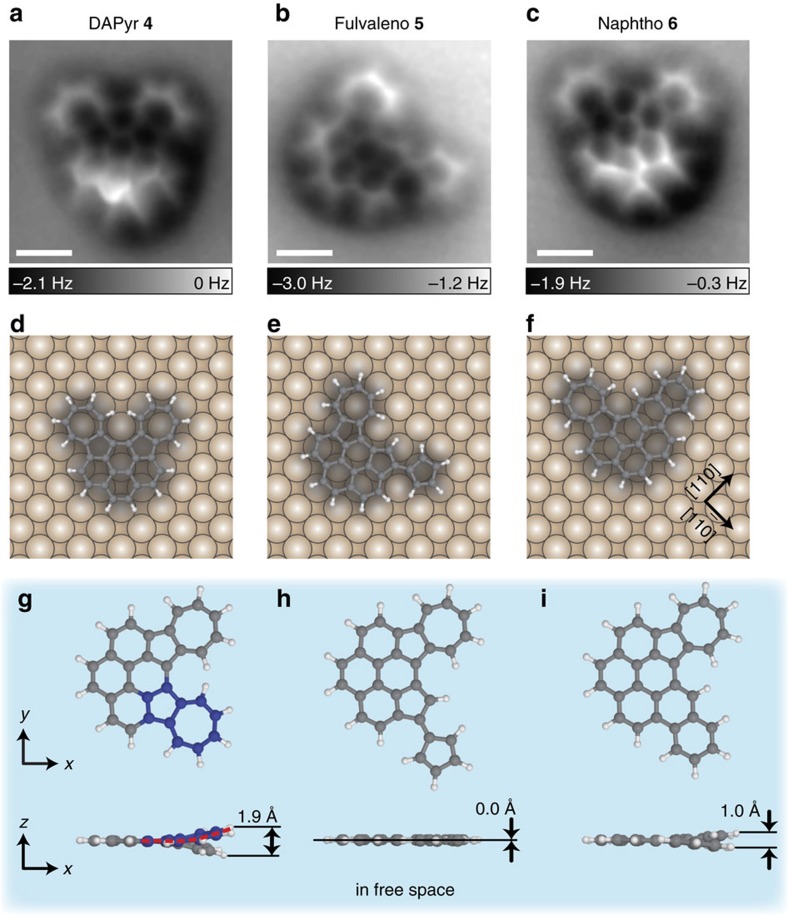
AFM images of the thermal reaction products of DAPh/Cu(001). (**a**–**c**) AFM images of **4**, **5** and **6**, respectively, on Cu(001) in constant-height mode with CO-terminal tips. (**d**–**f**) Schematic illustration of the adsorption structures of **4**, **5** and **6**, respectively. All of the products are expected to be flattened on the surface. (**g**–**i**) Calculated structures of **4**, **5** and **6**, respectively, in free space. Blue spheres in **g** represent C atoms of one of the azuleno moieties in DAPyr **4**. A bent red line in **g** indicates that the sterically hindered azuleno moieties are distorted in free space. The images in **a**–**c** were measured at the tip height corresponding to *V*=50 mV and *I*=200 pA over a bare surface. Scale bar, 5 Å.

High-resolution AFM imaging with CO-terminal tips is a useful technique for identifying the structure of the reaction intermediates and products. Figure 4a–c shows AFM images of **4**–**6**, respectively. As with **1** (Fig. 2c), all of the C–C bonds in each molecule are visible, suggesting that products **4**–**6** lie on the surface flatly. The AFM images determine the molecular structures and the reaction pathway as shown in Fig. 3e. The intermediate **4**, observed at *T*_anneal_≈130–210 °C, is assigned to a cyclodehydrogenated DAPh, namely diazuleno[1,2,3-*cd*:1′,2′,3′-*fg*]pyrene (DAPyr; see Fig. 4d). Figure 4b,c shows AFM images of **5** and **6**, respectively. The image indicates obviously that **5** (**6**) is yielded by the rearrangement of one of the two azuleno moieties in DAPyr **4** into a fulvaleno (naphtho) moiety as shown in Fig. 4e,f.

## Discussion

As stated above, DAPh **1** on the surface shows (i) cyclodehydrogenation at ∼130 °C, followed by (ii) the skeletal rearrangement above 200 °C, whereas DAA **2** is nonreactive. First, we consider the dehydrogenation mechanism. Although both DAPh **1** and DAA **2** are similarly distorted in free space (Fig. 1a,b), the sterically repelled H atoms of the former molecule can be dissociated thermally on the surface. The flattening energy of DAPh **1** is rather smaller than that of DAA 2 (Table 1), suggesting that the structural distortion does not directly contribute to this reaction. The reactivity difference is probably ascribable to the formability of the additional C–C covalent bonding. If DAA **2** was cyclodehydrogenated, the 5- and 6-positioned C atoms (Fig. 1b) would be bonded covalently to form a new pentagonal ring; however, this product is significantly unstable because the *sp*^2^-carbon skeleton is highly deformed. DAPh **1** is cyclodehydrogenated sensibly to form an additional hexagonal ring, but this reaction creates a large intramolecular distortion on the surface. The cyclodehydrogenated product—DAPyr **4**—is intrinsically twisted in free space because of steric hindrance between the azuleno moieties (Fig. 4g). As compared to DAPh **1** in which the phenanthrene part is twisted but the azuleno moieties are planar themselves (blue spheres aligning on a linear red line in Fig. 1a), the azuleno moieties of DAPyr **4** are distorted in free space (blue spheres aligning on a bent line in Fig. 4g) because of the rigid structure of pyrene part. Notably, for DAPyr **4**, the new six-membered carbon ring in the pyrene part has no aromatic feature, which was confirmed by calculations of nuclear independent chemical shift for the free molecules (see Supplementary Fig. 5). Therefore, this cyclodehydrogenated reaction does not provide the extension of the aromatic system. DAPyr **4** is flattened on the surface (Fig. 4d), and then, the distortion of the azuleno moieties gives rise to larger structural strain than that of the reactant **1** (Table 1). We assume that the structural strain localized at the azuleno moieties drives the skeletal rearrangement of **4**, as discussed below.

Species **3**, which appears before yielding **4**, probably intermediates the cyclodehydrogenated reaction from **1** to **4**. An AFM image of **3** (inset of Fig. 3d) shows an asymmetric outline with a high-contrast (that is, strongly repulsive) region located near the molecular centre. The sterically protruding structure of **3** complicates the determination of the detailed structure only from this image. We tentatively ascribed the intermediate to a DAPh with a C–C bond between the 12 and 13 positions without dehydrogenation (Fig. 3e). This molecule probably retains its aromaticity (26 π electrons) but is metastable because of the extremely distorted structure. However, we cannot exclude the possibility that species **3** includes a Cu adatom (that is, DAPyr-Cu complex); on-surface reactions can form metal–organic coordination complexes^56^ and AFM imaging has difficulty resolving the coordinated metal adatoms^42^,^57^.

Next, we discuss the skeletal rearrangement of DAPyr **4**. It is noteworthy that the product yield of **5** is far larger than that of **6**: 85 versus 2% at *T*_anneal_=245 °C (see Fig. 3d). In other words, an azuleno moiety of DAPh on Cu(001) is converted predominantly into a fulvaleno moiety, whereas a conversion into a naphtho moiety rarely occurs. Such selective rearrangement of azulene derivatives will play an important role in the controllable creation and repair of Stone–Wales defects in graphene structures^49^,^50^. To our knowledge, in general, both azuleno and fulvaleno moieties are converted into a naphtho moiety by heat^47^,^48^,^49^,^50^,^58^ and no azulene-to-fulvalene rearrangement has been reported. This strongly indicates that the interaction with the substrate plays an important role in yielding the specific reaction product. Furthermore, previous experimental and theoretical studies have revealed that various reaction mechanisms coexist for the rearrangement of azulene to naphthalene in the gas phase^48^,^49^,^50^. This suggests that multiple, coexisting reaction pathways are also conceivable for the reaction of **4** to **5**/**6** on the surface. Based on the previous reports for the azulene-to-naphthalene rearrangement, we propose several possible reaction mechanisms for **4**→**5**/**6** (see Supplementary Note 3 and Supplementary Figs 6 and 7).

Nonetheless, we can understand the mechanism of the selective reaction of **4**→**5** in a qualitative manner, by considering the correlation between the steric distortion and the reactivity of the molecules. In free space, as described above, the azuleno moieties of **4** are locally distorted by their mutual proximity (a molecular height of 2.2 Å; Fig. 4g), leading to a flattening energy of 171 meV at a reaction temperature of 227 °C (Table 1). A final product of the fulvaleno species **5** is planar even in free space (Fig. 4h). Therefore, the on-surface reaction of the distorted species **4** to yield the intrinsically planar product **5** proceeds exothermically to eliminate the strain instability in the molecule on the surface. The complete resolution of the geometric distortion may also be responsible for the fact that no reaction product with both azuleno moieties rearranged has been observed in our experiments. In contrast, the other product, that is, the naphtho species **6**, is still nonplanar (a molecular height of 1.0 Å and a flattened energy of 110 meV; Fig. 4i). The reaction of **4**→**6** is unfavourable because it retains the intramolecular strain, and **6** may be rearranged again into the totally planar product **5**.

Finally, we must also consider DAA **2**, which cannot be rearranged. The flattening energy of DAA **2** (104 meV at 227 °C; Table 1) is lower than that of DAPyr **4**. Moreover, even though one of the azuleno moieties of DAA is rearranged into a fulvaleno moiety, this product should retain the intramolecular strain with a flattening energy is 86 meV at 227 °C (see Supplementary Note 2 and Supplementary Fig. 8). We should note that the order of the flattening energies may be much smaller than the reaction barrier for the skeletal rearrangement; indeed, the barrier for the azulene-to-naphthalene rearrangement in the gas phase is calculated to be above 1 eV (refs 49, 50). We assume that the strain energy of DAPyr **4** is enhanced due to interactions with the substrate such as charge transfer. To gain further insights into the reaction mechanism, theoretical calculations of the adsorption structures and reaction barriers would be helpful.

In summary, we have demonstrated on-surface skeletal rearrangement of an azulene-derivative PAH driven by the intramolecular structural strain. To derive a highly strained PAH, we conducted a sequential procedure: organic synthesis of a PAH with two azuleno moieties in proximity to each other—that is, DAPh **1**—and thermal cyclodehydroganation of the molecule **1** adsorbed onto a Cu(001) surface. The product DAPyr **4** adopts a planar configuration on the surface, in spite of the fact that the azuleno moieties are sterically hindered by further mutual proximity. Therefore, the azuleno moieties flattened on the surface are highly strained. In order to eliminate the local strain instability, the molecule **4** is thermally induced to react predominantly into an intrinsically flat PAH with a fulvaleno moiety, which has never been yielded by conventional organic synthesis. The importance of the strain for the skeletal rearrangement is reinforced by the observation that a reference molecule—DAA **2**—exhibits no thermal reactivity at the surface because it cannot eliminate the strain by any skeletal rearrangement. Such strain-induced reactions of organic molecules on surfaces would be a useful approach to synthesizing functional chemical products.

## Methods

### Experiments

The STM/AFM experiments were carried out in an ultrahigh-vacuum chamber (Omicron low-temperature STM/AFM system) at 4.8 K. For frequency-modulation AFM, a tuning fork with an etched tungsten tip was used as a force sensor^59^ (resonance frequency *f*_0_=21.4 kHz, spring constant *k*_0_≈1,800 N m^−1^, quality factor *Q*≈2 × 10^4^). For the AFM images, Δ*f* was measured in constant-height mode at a sample bias *V*=0 V and an oscillation amplitude *A*=2 Å, while the STM images were acquired in constant-current mode. Single-crystalline Cu(001) was cleaned by repeated cycles of Ar^+^ sputtering and annealing.

The methods of synthesis, purification and characterization for DAPh **1** and DAA **2** are described in Supplementary Methods and Supplementary Figs 9–16. These molecules were thermally sublimated from alumina crucibles at ∼130 °C and deposited onto a clean surface at room temperature. The surface at ∼8 K was subsequently exposed to CO gas.

### Calculations

The geometric optimizations of the molecules in free space were performed by Materials Studio 2016 DMol^3^ B3LYP functional with a DNP basis set. The details of the calculations are described in Supplementary Methods.

### Data availability

Supplementary crystallographic information files, which include structure factors, have been deposited with the Cambridge Crystallographic Data Centre (CCDC) as deposition numbers CCDC 1522349, **1**; CCDC 1,522,350, **2**. These data files can be obtained free of charge from http://www.ccdc.cam.ac.uk/data_request/cif. The data that support the findings of this study are available from the corresponding author on reasonable request.

## Additional information

**How to cite this article:** Shiotari, A. *et al*. Strain-induced skeletal rearrangement of a polycyclic aromatic hydrocarbon on a copper surface. *Nat. Commun.* **8,** 16089 doi: 10.1038/ncomms16089 (2017).

**Publisher’s note:** Springer Nature remains neutral with regard to jurisdictional claims in published maps and institutional affiliations.

## Supplementary Material

Supplementary Information

Supplementary Date 1

Supplementary Data 2

## Figures and Tables

**Table 1:** Calculated flattening energies in free space.

	**DAPh 1**	**DAA 2**	**DAPyr 4**	**Fulvaleno 5**	**Naphto 6**
Δ*G* (25 °C)	0.014	0.045	0.098	0.000	0.058
Δ*G* (227 °C)	0.060	0.104	0.171	0.000	0.110

The energies are calculated in eV. Δ*G*(*T*) represents the Gibbs energy difference between the free molecule *G*_free_ and the planar molecule (located in the *x*–*y* plane with *z* restricted to zero) *G*_planar_ at a temperature *T*, that is, Δ*G*=*G*_planar_−*G*_free_.
