# Supplementary material for: Strain-induced skeletal rearrangement of a polycyclic aromatic hydrocarbon on a copper surface
Source: Nat Commun. 2017 Jul 20;8:16089. doi: 10.1038/ncomms16089 (PMC5524995; doi:10.1038/ncomms16089)
Supplement: Supplementary Information [file ncomms16089-s1.pdf]

Title of file for HTML: Supplementary Information
Description: Supplementary Figures, Supplementary Notes, Supplementary Methods and Supplementary References

Title of file for HTML: Supplementary Date 1
Description: Single crystal structure for DAPh

Title of file for HTML: Supplementary Data 2
Description: Single crystal structure for DAA

Supplementary Figures

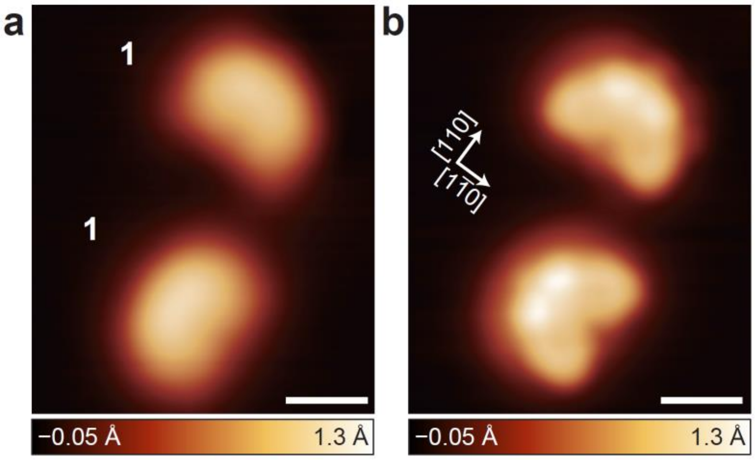

Supplementary Figure 1 | STM images of diazuleno[1,2-*c*:2',1'-*g*]phenanthrene (DAPh) on Cu(001) with Cu- and CO-terminal tips. a,**b**, STM images of DAPh with Cu- and CO-terminal tips, respectively. The images were obtained with the sample bias *V* = −30 mV and the tunnelling current *I* = 10 pA. Scale bar, 10 Å.

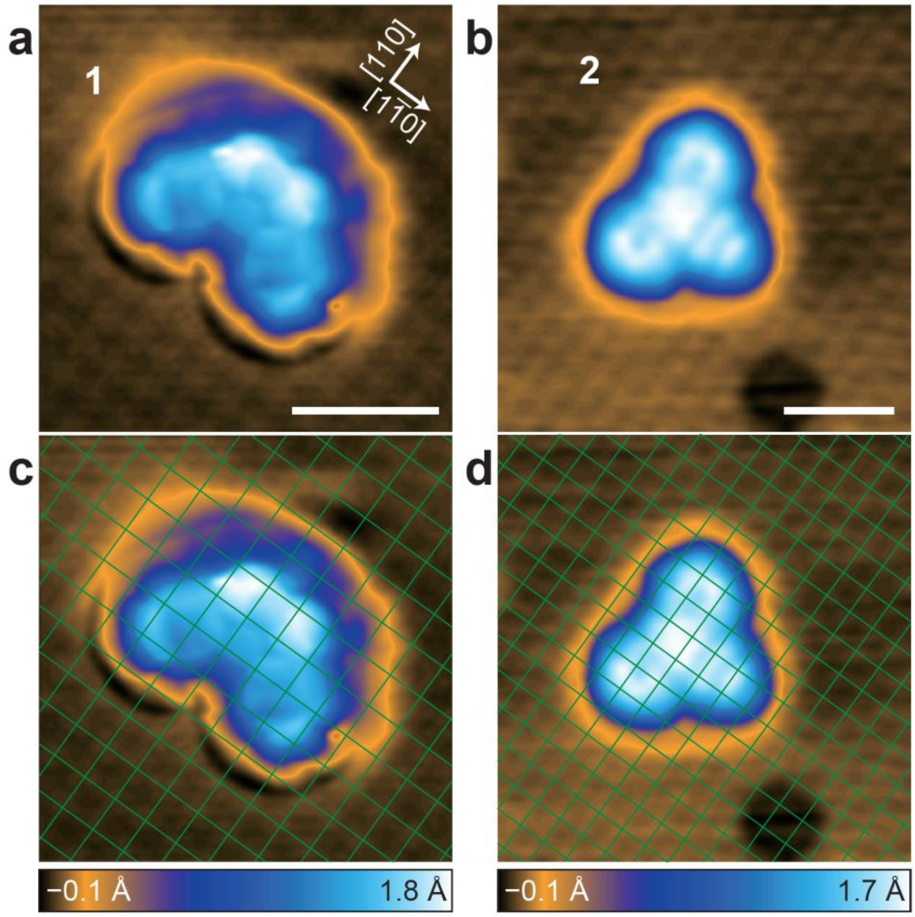

Supplementary Figure 2 | STM images of DAPh and DAA with surface Cu atoms. **a**,**b**, STM images of DAPh and DAA, respectively, together with surface Cu atoms. **c**,**d**, The same as **a** and **b**, respectively, but with the green lines representing the lattice of the Cu atoms (the unit cell is 2.56 Å × 2.56 Å). The image in **a** (**b**) was obtained with V = 50 mV and I = 2 (0.2) nA. Scale bar, 10 Å.

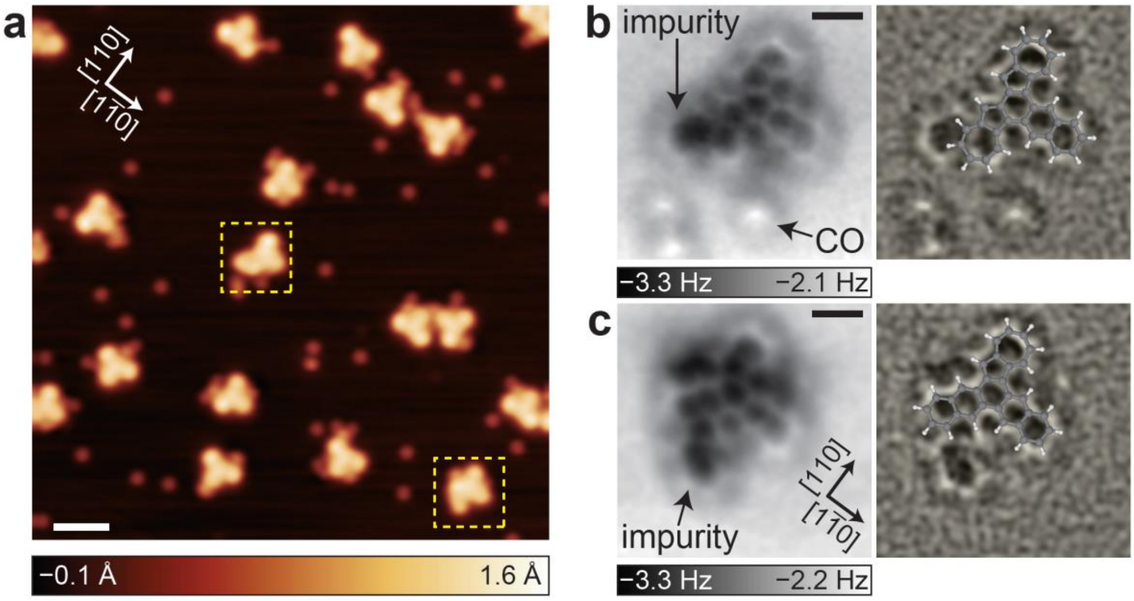

Supplementary Figure 3 | STM images of DAA/Cu(001) annealed at 275 °C. a, STM images of DAA with CO terminal tips after the sample was annealed at 275 °C for 10 min. **b**,**c**, AFM images of the regions surrounded by the upper and lower yellow-dotted squares, respectively, in **a**. Laplace-filtered AFM images together with the molecular structures are also shown on the right side. The image in **a** was obtained with *V* = 50 mV and *I* = 20 pA. The images in **b** and **c** were acquired with *V* = 0 V at a constant tip height corresponding to *V* = 50 mV and *I* = 20 pA over a bare surface. Scale bars, 20 Å (**a**); 5 Å (**b**,**c**).

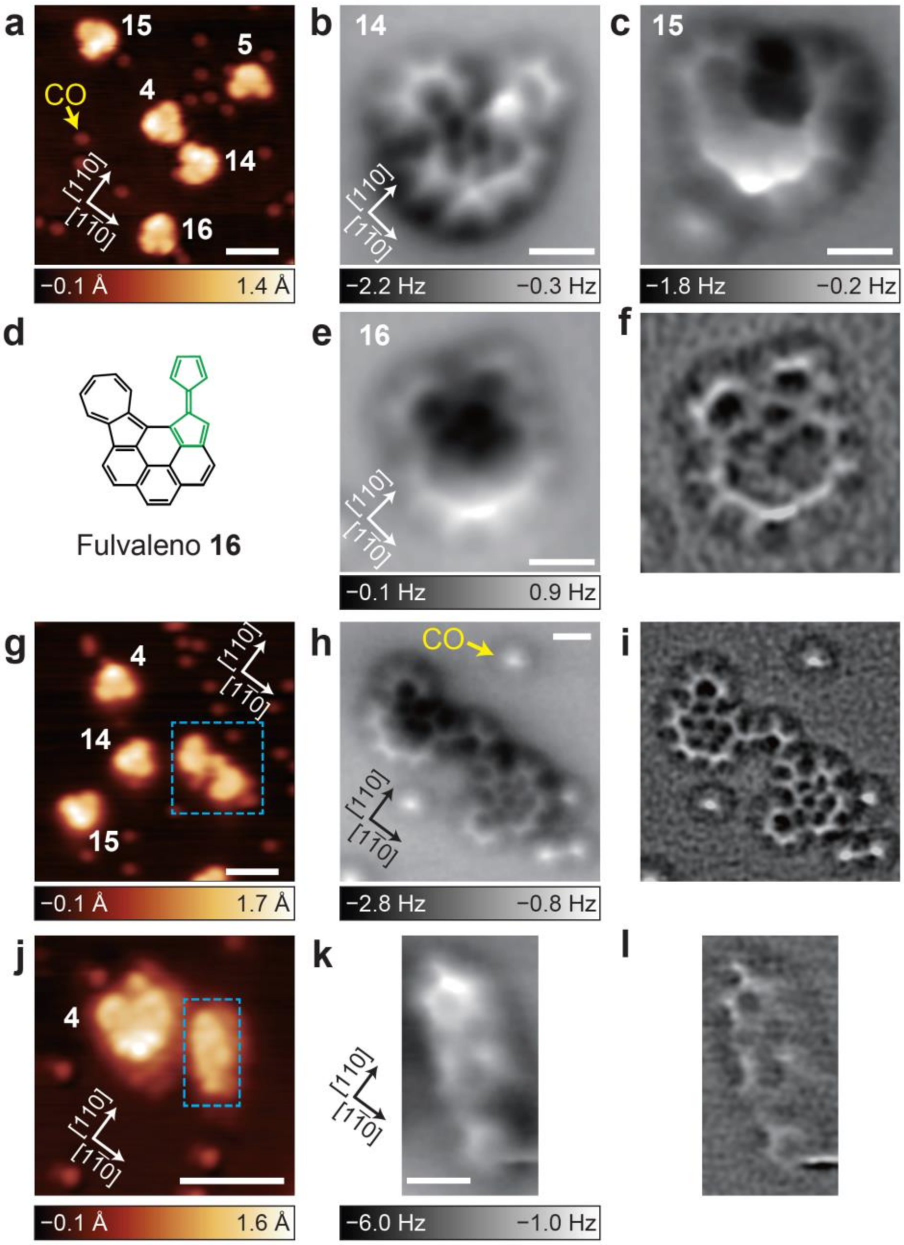

Supplementary Figure 4 | Minor species of DAP/Cu(001) | a,**g**,**j**, STM images of DAPh/Cu(001) at T_{anneal} = 165 °C with CO terminal tips. **b**,**c**,**e**, AFM images of species **14–16**, respectively. **d**, Proposed structure of **16**, which is a minor product with a fulvaleno moiety. **h**,**k**, AFM images within the blue box in **g** and **j**, respectively. **f**,**i**,**l**, Laplace-filtered AFM images of **e**, **h**, and **k**, respectively. The images were acquired with the following parameters: V = 50 mV and I = 20 pA for **a** and **g**, V = 50 mV and I = 200 pA for **j**, V = 0 mV and Δz = 0 Å for **b** and **h**, V = 0 mV and Δz = +0.5 Å for **c**, V = 0 mV and Δz = +0.8 Å for **e**, V = 0 mV and Δz = −0.5 Å for **k**. The origin of Δz is defined as the tip height corresponding to V = 50 mV and I = 200 pA over a bare surface. Scale bars, 20 Å (**a**,**g**,**j**); 5 Å (**b**,**c**,**e**,**h**,**k**).

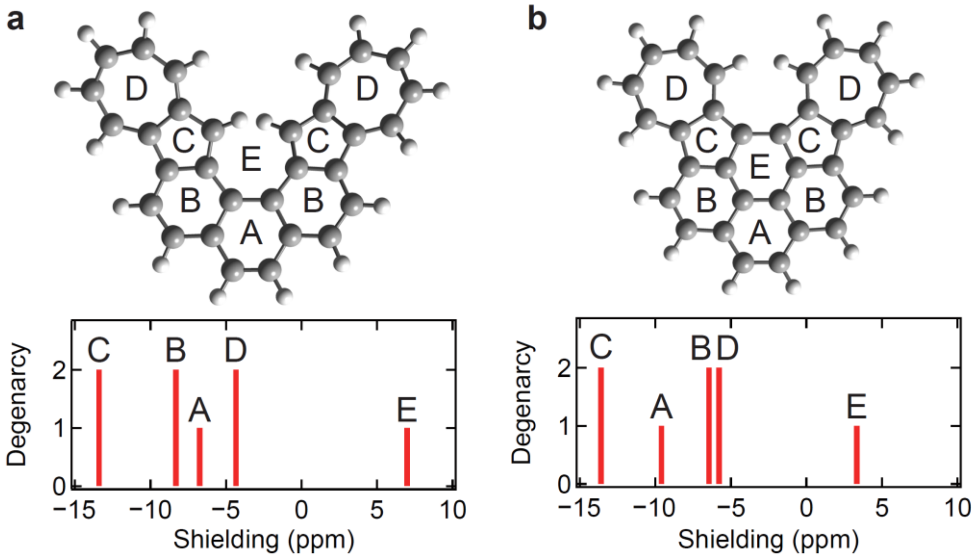

Supplementary Figure 5 | Nuclear independent chemical shift (NICS) calculated for free molecules. a, DAPh **1** and **b**, DAPyr **4**. NICS evaluates the absolute magnetic shielding at the centre of each carbon ring system[1]; negative NICS values indicate aromaticity (*e.g.* −9.7 for benzene[1]) whereas positive values indicate antiaromaticity. Position E represents the centre of the six carbon atoms that form a new six-membered ring by the cyclodehydrogenation of DAPh **1** to DAPyr **4**. The NICS values at position E are +6.99 for **1** and +3.33 for **4**, indicating that the carbon ring has no aromatic feature, even after the cyclodehydrogenation.

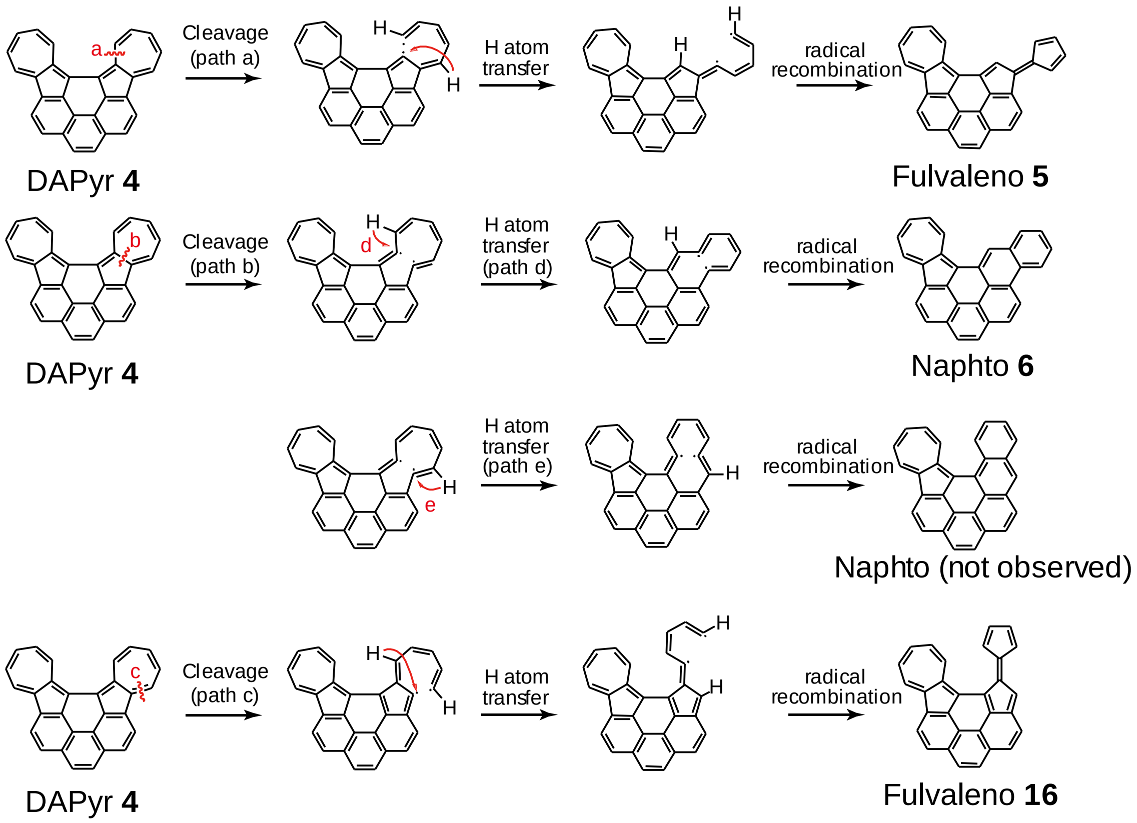

Supplementary Figure 6 | Possible reaction pathways of the rearrangement of DAPyr 4 mediated by open-ring reactions.

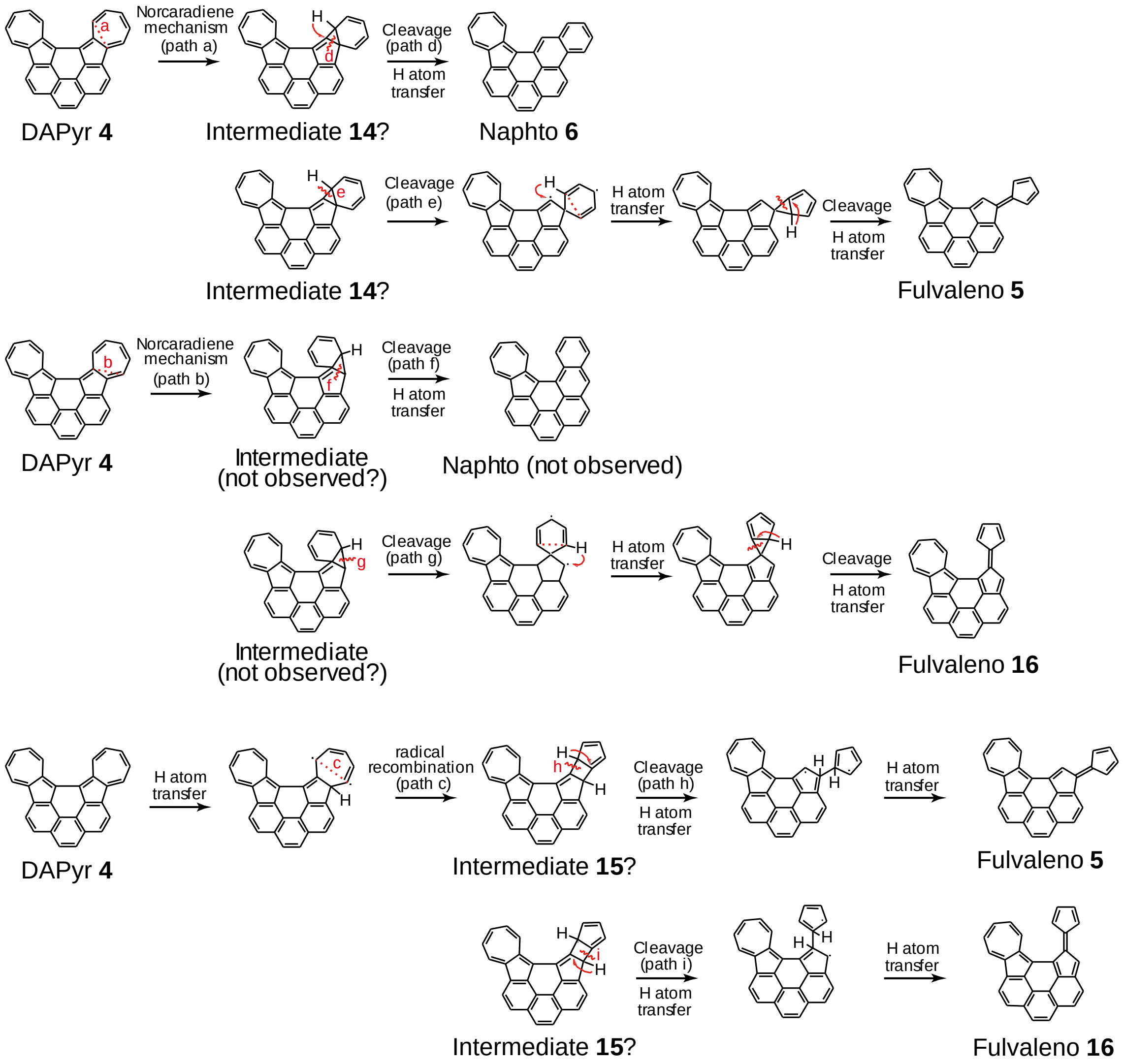

Supplementary Figure 7 | Possible reaction pathways of the rearrangement of DAPyr 4 mediated by the norcaradiene mechanism.

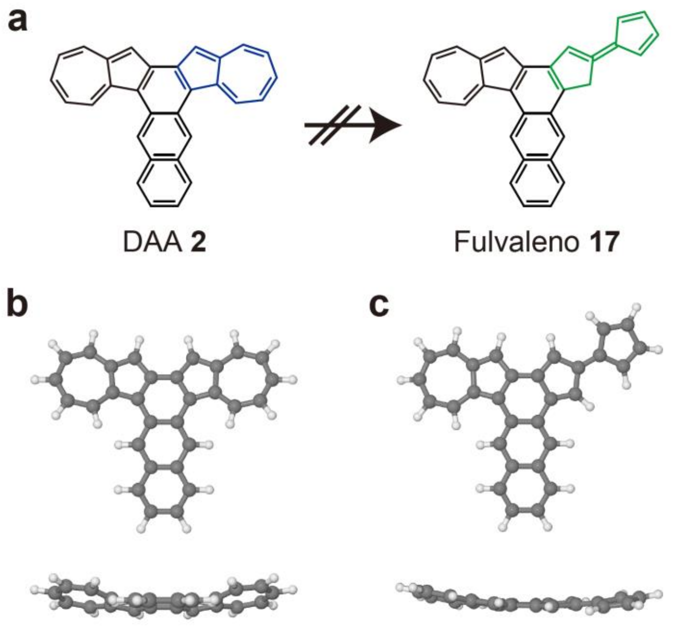

Supplementary Figure 8 | Assumption of the fulvaleno-rearranged species of DAA 2. a, Scheme of an assumed skeletal rearrangement of DAA **2** to yield a fulvaleno species **17**. **b**,**c**, Calculated structures of **2** and **17**, respectively.

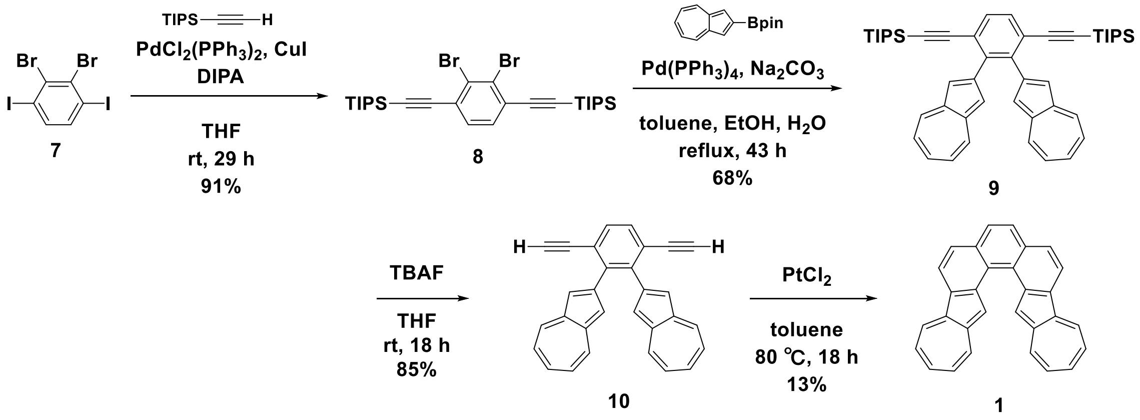

Supplementary Figure 9 | Synthetic route of diazuleno[1,2-*c*:2',1'-*g*]phenanthrene (1).

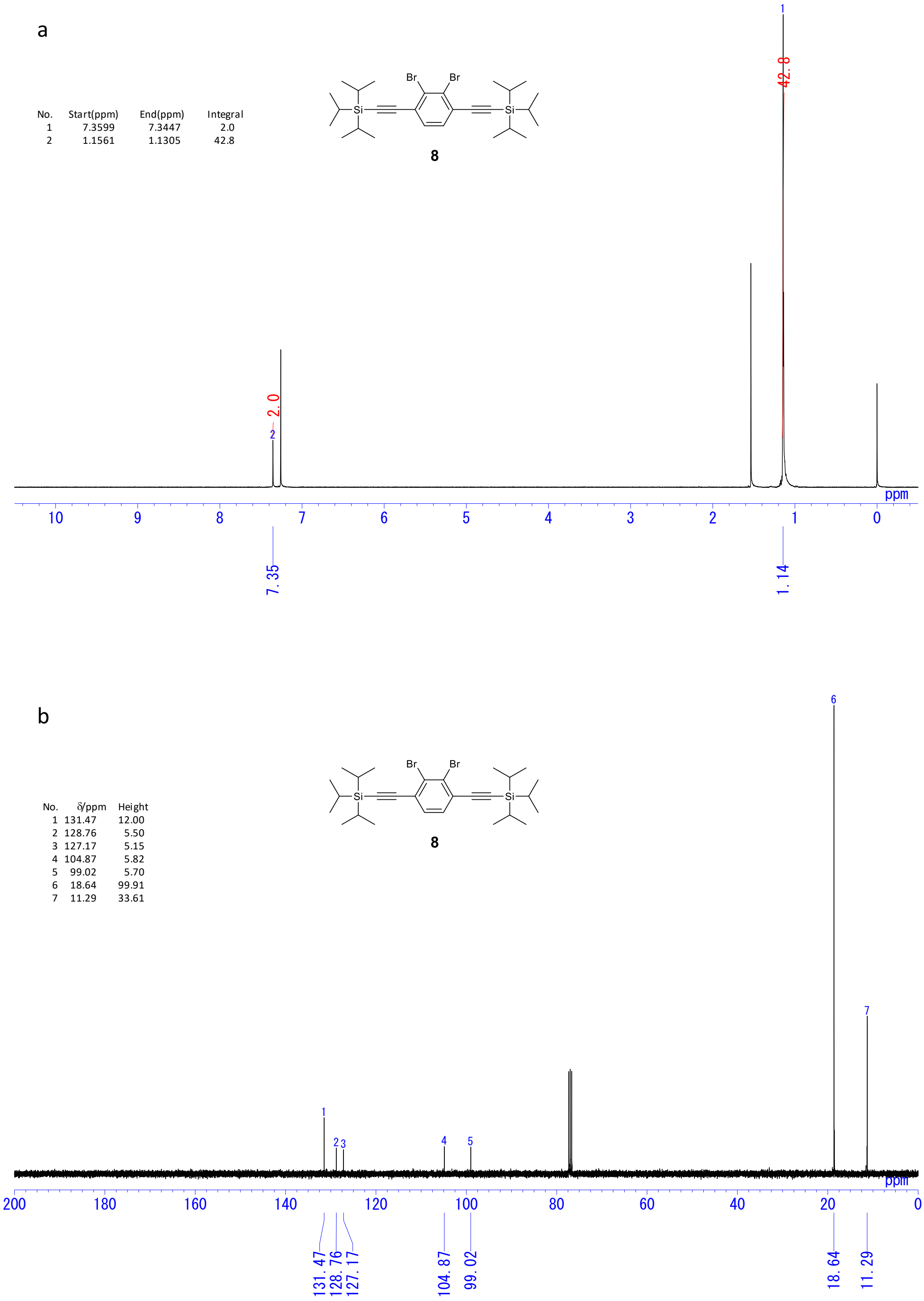

Supplementary Figure 10 | NMR spectra of 8 in $CDCl_3$. a, ^{1}H NMR; **b**, ^{13}C NMR.

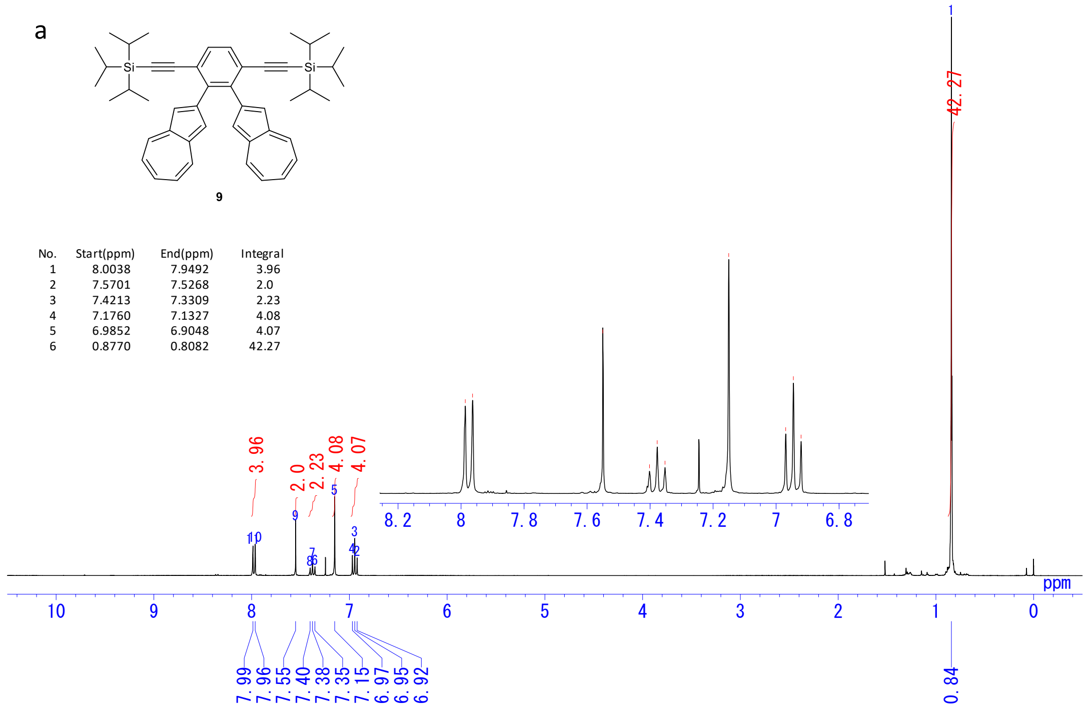

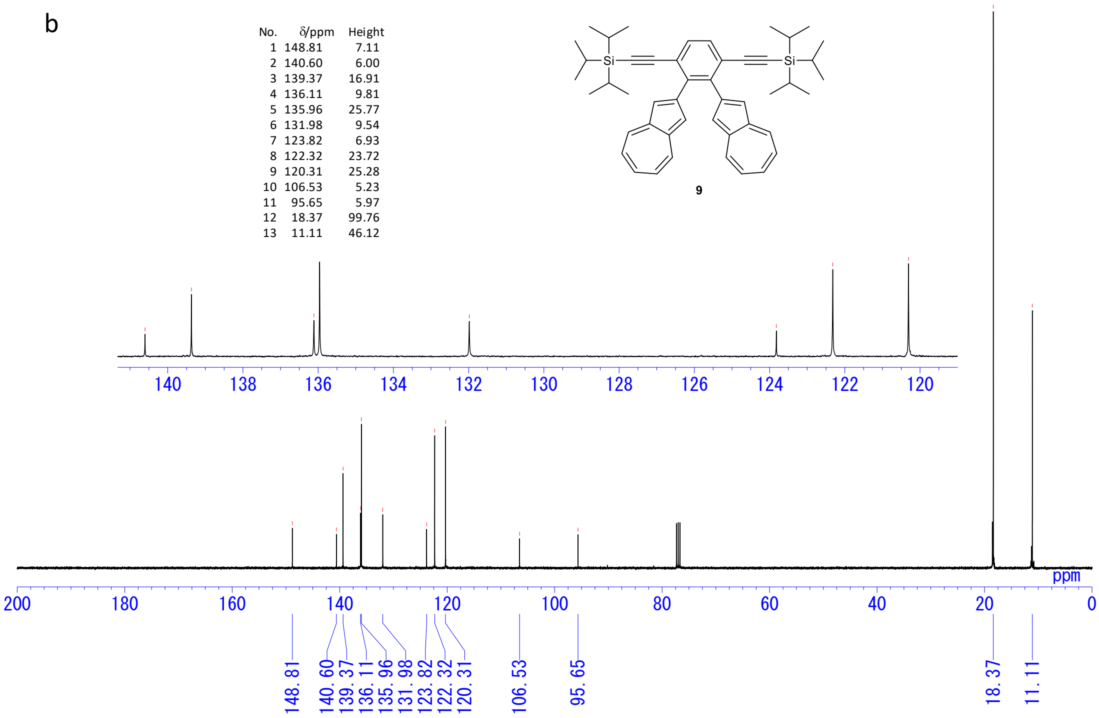

Supplementary Figure 11 | NMR spectra of 9 in $CDCl_3$. a, ^{1}H NMR; **b**, ^{13}C NMR.

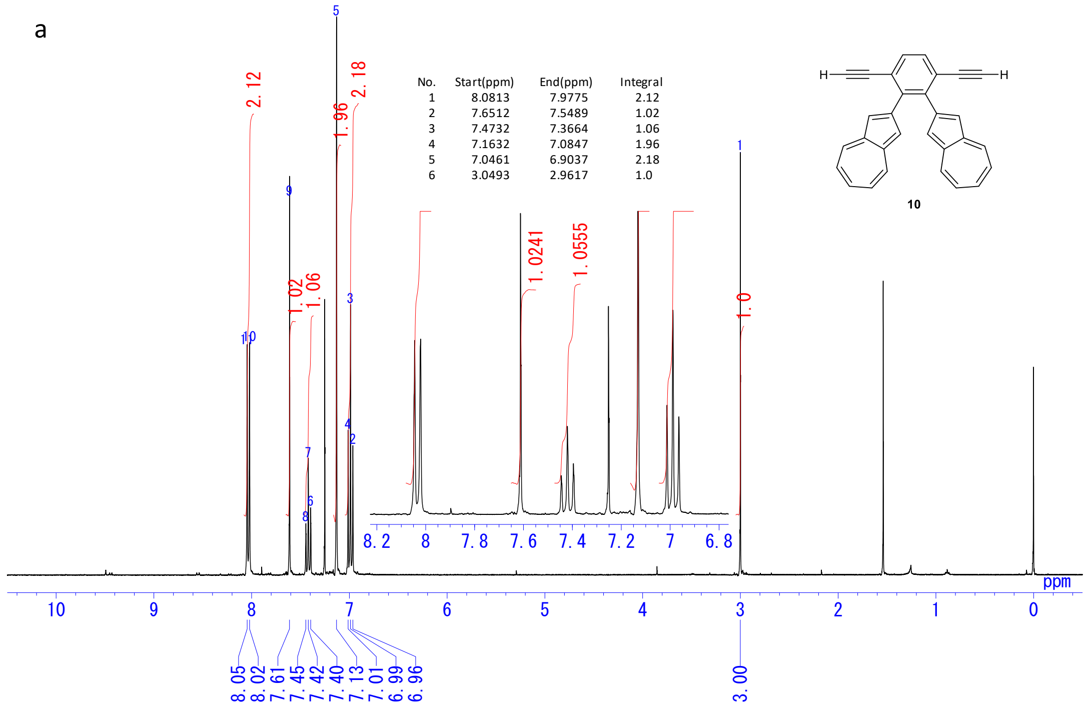

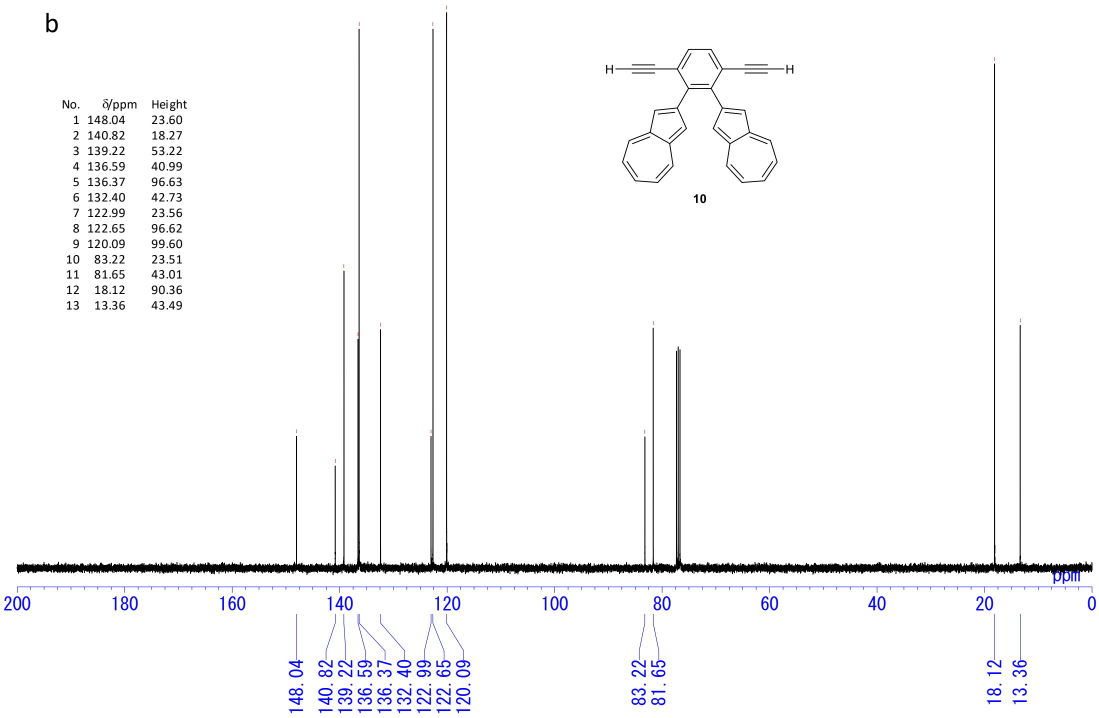

Supplementary Figure 12 | NMR spectra of 10 in $CDCl_3$. a, ^{1}H NMR; **b**, ^{13}C NMR.

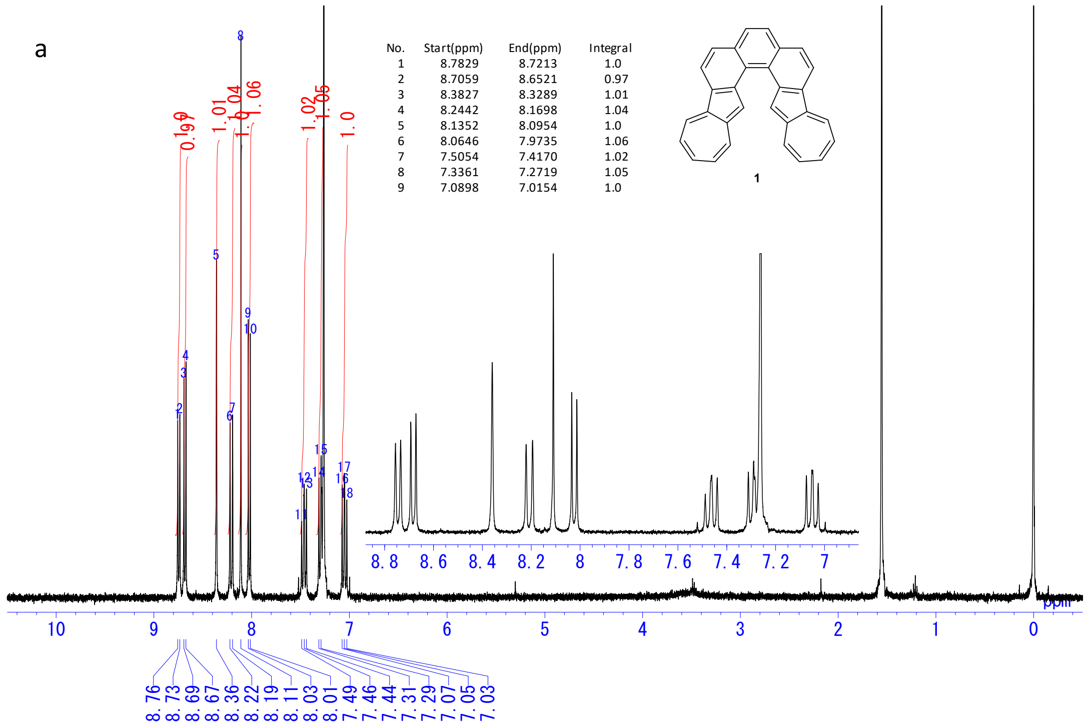

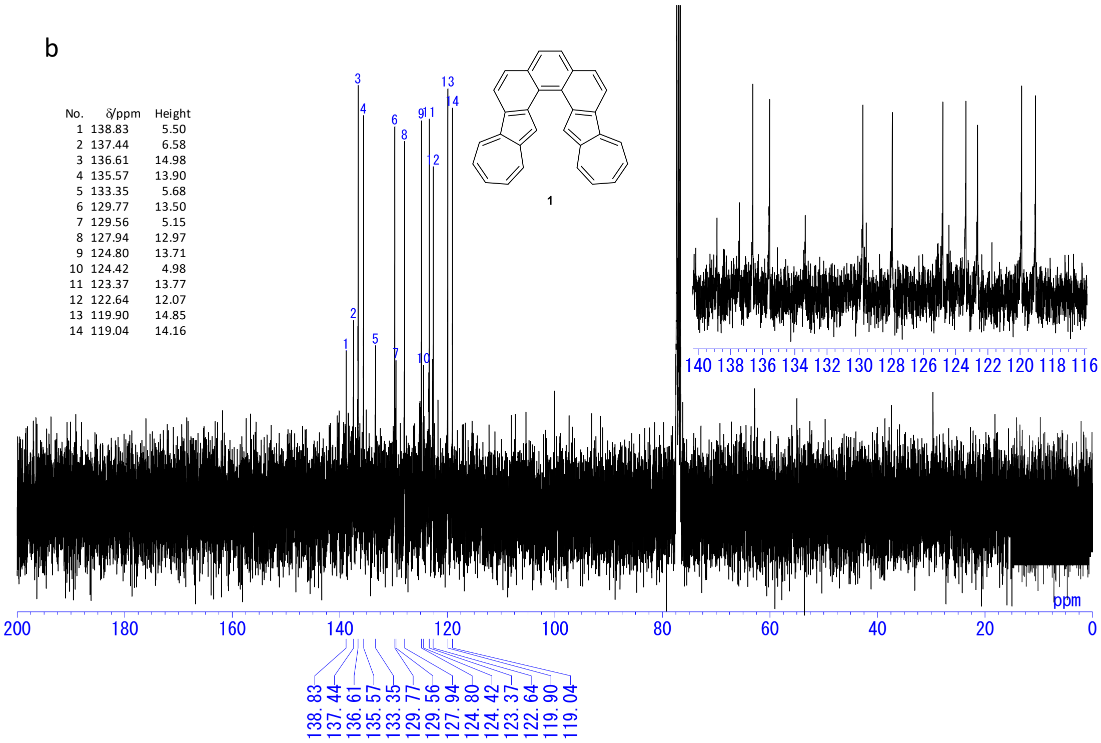

Supplementary Figure 13 | NMR spectra of 1 in CDCl$_3$. a, ^{1}H NMR; **b**, ^{13}C NMR.

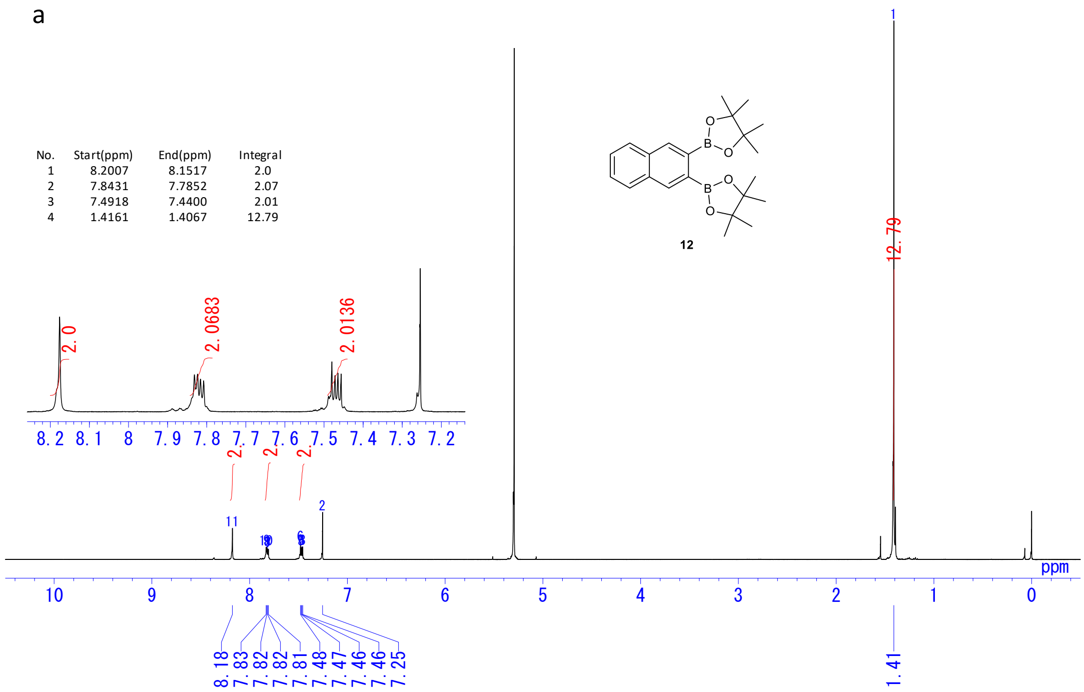

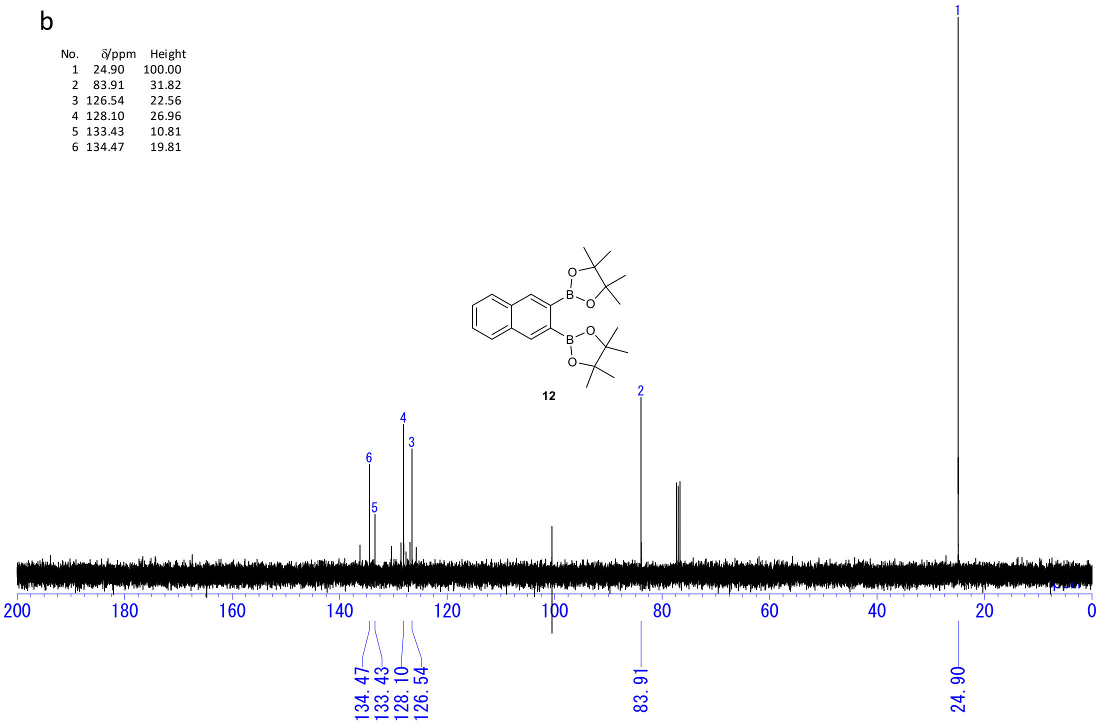

Supplementary Figure 14 | NMR spectra of 12 in $CDCl_3$. a, ^{1}H NMR; **b**, ^{13}C NMR.

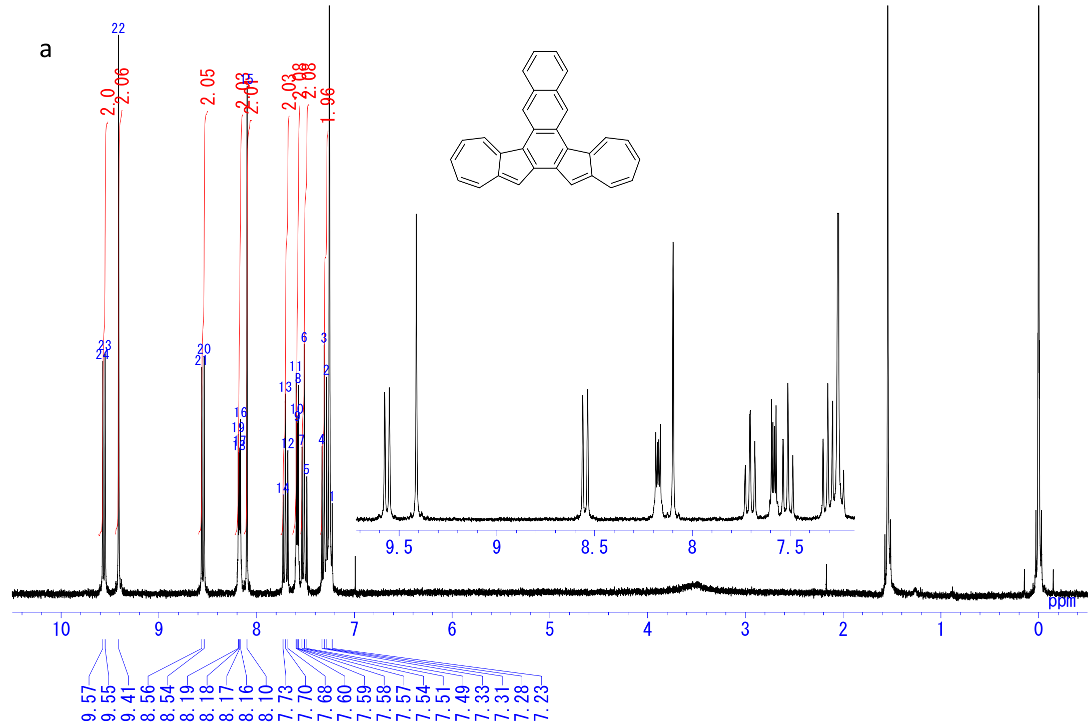

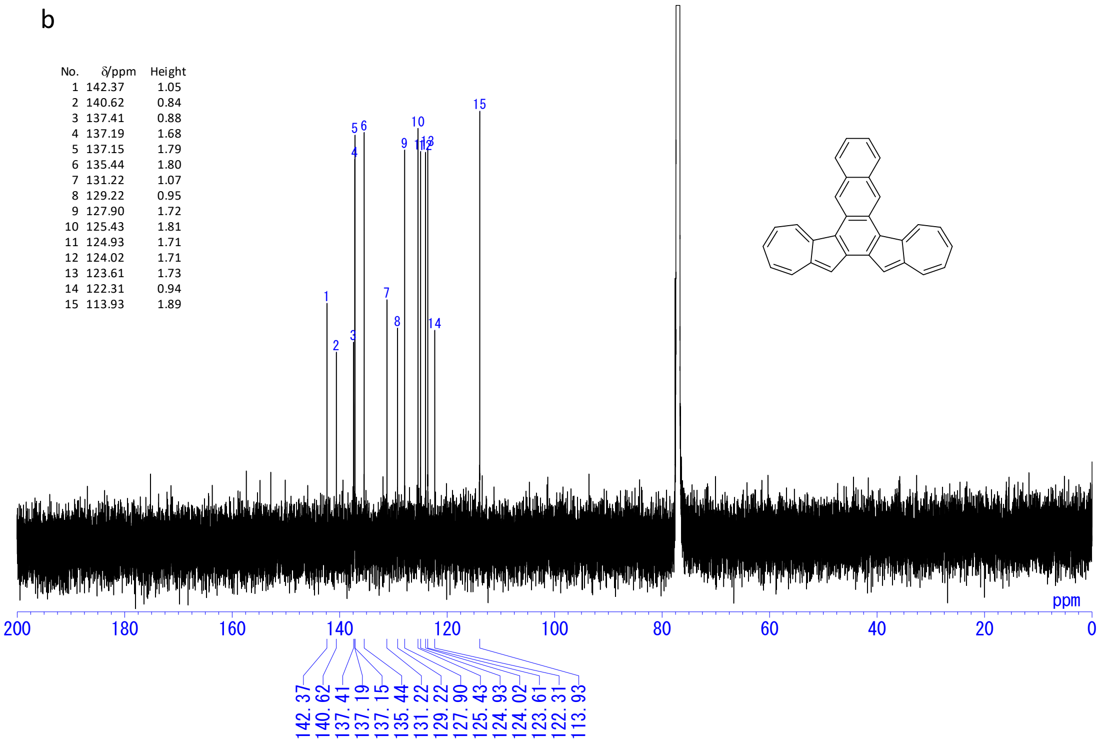

Supplementary Figure 15 | NMR spectra of 2 in $CDCl_3$. a, ^{1}H NMR; **b**, ^{13}C NMR.

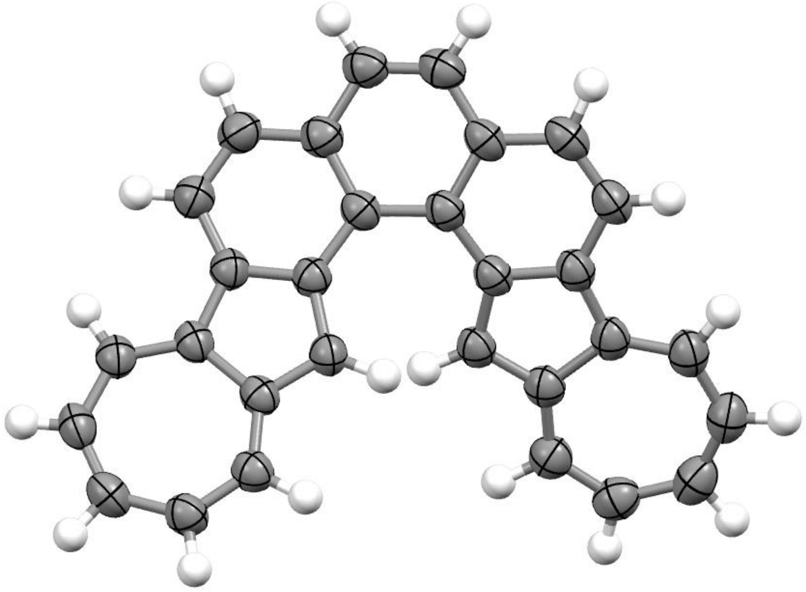

1

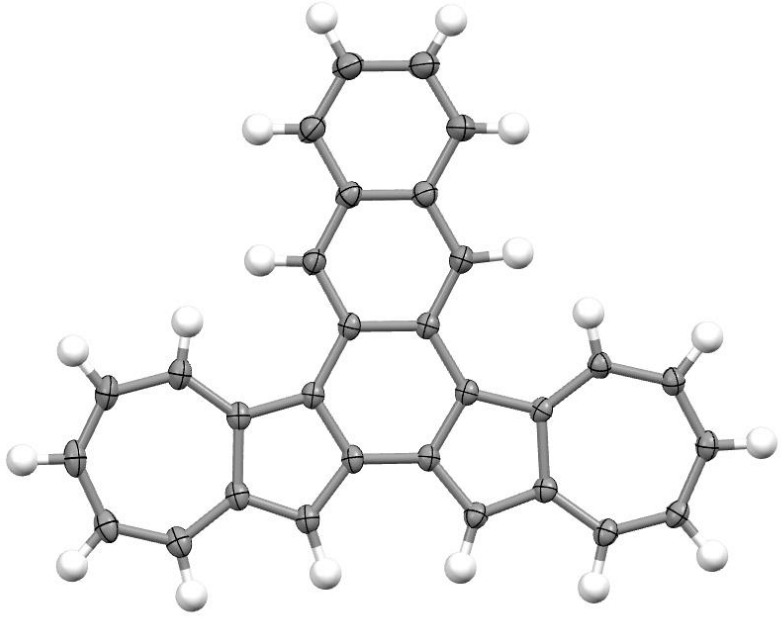

2

Supplementary Figure 16 | Crystallographically determined structures of 1 and 2. Ellipsoids represents 50% probability.

Supplementary Notes

Supplementary Note 1: Additional STM images of DAPh 1

Supplementary Figure 1a shows typical STM images of diazuleno[1,2-*c*:2',1'-*g*]phenanthrene (DAPh) molecules on Cu(001) with a Cu-terminal tip. The tip was prepared by poking the apex into the surface. The molecules are observed as bean-shaped protrusions. By attaching a CO molecule to the tip apex, the detailed structure can be resolved (Supplementary Fig. 1b).

Supplementary Figure 2a,b shows STM images of DAPh and diazuleno[1,2-*a*:2',1'-*c*]anthracene (DAA), respectively, on Cu(001) with CO-terminal tips. The molecules and surface Cu atoms are observed simultaneously, and thus the adsorption sites of these molecules can be determined as shown by the green lines in Supplementary Fig. 2c,d. We note that the inner features of the images probably reflect the shape of the molecular frontier orbitals, but no resonance state was observed in the dI/dV spectra of the molecules in the sample-bias range from $V = -2$ to 2 V (with Cu-terminal tips).

Supplementary Note 2: Additional experimental and calculation data for DAA 2

Supplementary Figure 3a shows STM images of DAA on Cu(001) at T_{anneal} = 275 °C. Most of the molecules are observed as T-shaped protrusions, indicating the DAA molecules are not reacted. No isolated molecule with a rearranged azuleno moiety was observed. On the other hand, a few images, surrounded by the yellow squares in Supplementary Fig. 3a, apparently have different shapes. The molecular structures in these image were confirmed with AFM (Supplementary Fig. 3b,c). The molecule in Supplementary Fig. 3b (upper square in Supplementary Fig. 3a) seems to remain a pristine structure, *i.e.*, DAA, but an impurity is attached to it. The other molecule in Supplementary Fig. 3c (bottom square in Supplementary Fig. 3a) is probably assigned to an azuleno-to-naphtho rearranged molecule (azuleno[1,2-*r*]pentaphene) with an impurity. Molecules accompanied by an impurity have various structures. The chemical compositions of the impurities are unknown, but we suggest that these originate from thermally dissociated DAA molecules because no similar structure was observed below the annealing temperature. Therefore, we conclude that no specific product was obtained.

We have not observed any specific skeletal rearrangements of DAA **2** on Cu(001). To confirm that this molecule is inert, we assume that the rearrangement of one of the azuleno moieties of DAA **2** into a fulvaleno moiety to yield the product **17** (Supplementary Fig. 8a). We calculated the optimized structure in free space, as shown in Supplementary Fig. 8c. Like the reactant **2** (Supplementary Fig. 8b), **17** remains nonplanar in free space. The flattening energy of

2 (**17**) is calculated to be 0.045 (0.067) eV at 25 °C and 0.104 (0.086) eV at 227 °C. This suggests that the intramolecular strain of the molecule is almost unchanged even after the rearrangement. Therefore, the reaction of **2** →**17** is expected to be unfavourable on the surface.

Supplementary Note 3: Additional AFM images of DAPh/Cu(001) and possible mechanisms of the reaction 4 → 5/6

Supplementary Figure 4a shows STM images of DAPh on Cu(001) at T_{anneal} = 165 °C. In addition to DAPyr **4** and the major product **5**, several kinds of species coexist. Supplementary Figure 4b,c,e shows the AFM images of species labelled as **14–16**, respectively. In each molecule, one of the azuleno moieties were reacted. The AFM images of the reacted moieties in **14** and **15** are higher protruded, indicating that the moieties are not flat but sterically distorted. We assume that these species are intermediates of the rearrangement of DAPyr **4** into **5** and/or **6**. In contrast, the species **16** (Supplementary Fig. 4e,f) is probably a fulvaleno-rearranged product as shown in Supplementary Fig. 4d. Because the abundance yield of **16** is about 2% (whereas 27% for **5** and 3% for **6**), **16** is a minor product.

We also observed another very minor species. Supplementary Figure 4g,h shows STM images of the different areas of the sample. The AFM images within the blue boxes in Supplementary Fig. 4h,k, respectively. For the two molecules in Supplementary Fig. 4h,i, one of the seven-membered rings is cleaved and are probably fused each other. This suggests that the rearrangement of **4** into **5** is mediated by the open-ring reactions. The ring-opened species were only observed when two molecules were located closely each other, implying that the reaction was prevented by the neighbouring molecule. The species in Supplementary Fig. 4k,l seems smaller than the reactant (DAPh **1**). Although the molecular structure is unclear, we assume that this species is an impunity or a dissociated product. The abundance yield of such fragments is less than 1%.

For the on-surface rearrangement of DAPyr **4**, we observed the intermediates (**14**, **15**, and ring-opened species; Supplementary Fig. 4b,e,h), the major product (fulvaleno **5**; Fig. 4b in the main text), and the minor products (naphtho **6** and fulvaleno **16**; Fig. 4c in the main text and Supplementary Fig. 4e). We propose several reaction pathways of the rearrangement, based on the previous reports for the rearrangement of azulene to naphthalene in the gas phase[2–4].

Supplementary Figure 6 shows the reaction pathways that are mediated by open-ring reactions. The difference of the cleaved C–C covalent bond causes multiple products including **5**, **6**, and **16**. Supplementary Figure 7 shows other possible reaction pathways, which is proposed by reference to the norcaradiene mechanism of the azulene-to-naphthalene rearrangement[2–4]. The intermediates in this mechanism are similar to the observed species **14** and **15**.

Supplementary Methods

Synthesis

All manipulations were performed under nitrogen using standard Schlenk techniques. NMR spectra were recorded on JEOL JNM-ALS400 (400 MHz for ^{1}H, 100 MHz for ^{13}C), instruments. NMR spectra were reported in parts per million using tetramethylsilane (δ 0.00 ppm for chloroform-d) or residual solvent (δ 7.26 ppm for chloroform-d) as the internal standard for ^{1}H NMR, and from the solvent carbon (δ 77.00 ppm for chloroform-d) for ^{13}C NMR. Mass spectra were recorded by JEOL JNM-MS700v for EI, and Applied Biosystems Voyager System 6194 for MALDI-TOF. Melting points were measured with a Yanaco M-500D melting point apparatus. Elemental analyses were performed on a Yanako CHN Corder MT-5 system at the Advanced Research Support Center, Ehime University. Thin-layer chromatography (TLC) and column chromatography were performed on Art. 5554 (Merck KGaA) and silica gel 60N (Kanto Chemical Co.), respectively. Gel permeation chromatography (GPC) was performed on an LC-9201 system (Japan Analytical Industry) with JAIGEL 1H and 2H polystyrene columns (eluent: $CHCl_3$, flow: 3.5 mL/min).

2,3-Dibromo-1,4-diiodobenzene (**7**)[5], 2,3-diiodonaphthalene (**11**)[6] and 1,1',3,3'-tetrabromo-2,2'-biazulene (**13**)[7] were prepared according to the literature. THF (anhydrous, without inhibitor grade) purchased from Kanto Chemicals was used under nitrogen. The other commercially available reagents were used as received, unless otherwise noted.

((2,3-Dibromo-1,4-phenylene)bis(ethyne-2,1-diyl))bis(triisopropylsilane): (8)

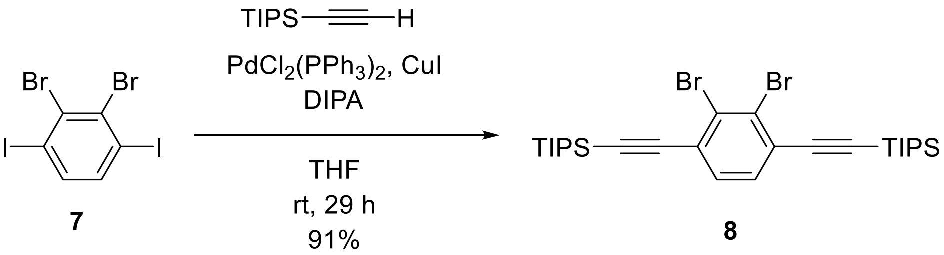

A 300-mL three-necked flask was equipped with a magnetic stirring bar, three-way stopcock connected with a vacuum-nitrogen manifold. 2,3-Dibromo-1,4-diiodobenzene (6.18 g, 12.7 mmol), $PdCl_2(PPh_3)_2$ (0.64 g, 0.91 mmol), CuI (0.39 g, 2.03 mmol), and diisopropylamine (16.0 mL) were placed in the reaction flask and degassed by three freeze-pump-thaw cycles. The mixture were diluted with THF (anhydrous and degassed, 130.0 mL), and stirred for 30 min at rt. To the mixture, triisopropylsilylacetylene (6.25 mL) was added dropwisely. The reaction mixture were stirred for further 22 hours at rt. The reaction was quenched by adding saturated NH_4Cl aqueous solution. The organic layer was washed with water (3 times), dried over Na_2SO_4, filtered

off the desiccant, and adsorbed on silicagel by concentration under vacuo. The crude material adsorbed on the silica gel was charged on a short silicagel pad and eluted with hexane to remove polar impurities. Obtained hexane fraction was concentrated by evaporation to obtain a solid material. Desired ((2,3-dibromo-1,4-phenylene)bis(ethyne-2,1-diyl))bis(triisopropylsilane) (**8**, 6.95 g, 92%) was obtained by washing with a minimum amount of methanol as a white solid.

^{1}H NMR ($CDCl_3$, 25 °C, 400 MHz) *δ* 7.35 (s, 2H, a), 1.16-1.13 (m, 42H, b); ^{13}C NMR ($CDCl_3$, 25 °C, 100 MHz) *δ* 131.47, 128.76, 127.17, 104.87, 99.02, 18.64, 11.29; MS (EI$^+$) 594.; HR MS (EI$^+$) calcd. 594.1348, observed 594.1342; Elemental Analysis Found C 56.37%, H 7.43%, observed C 56.29%, H = 7.51%.

((2,3-Di(azulen-2-yl)-1,4-phenylene)bis(ethyne-2,1-diyl))bis(triisopropylsilane): (9)

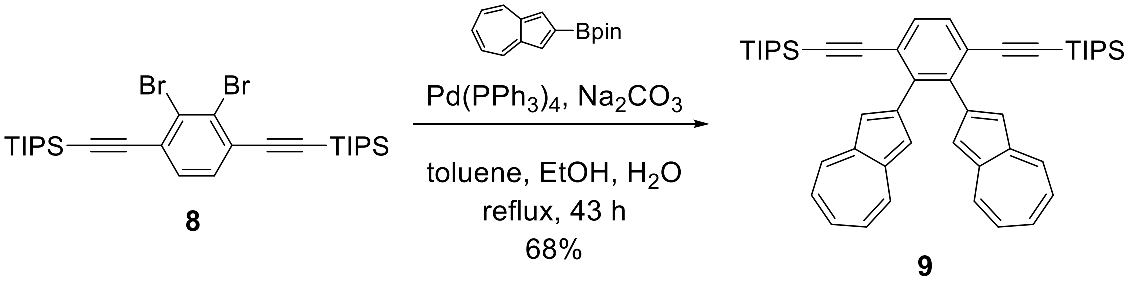

A 30-mL Schrenk flask was equipped with a magnetic stirring bar, three-way stopcock connected with a vacuum-nitrogen manifold. ((2,3-dibromo-1,4-phenylene)bis(ethyne-2,1-diyl))bis(triisopropylsilane) (**8**,100 mg, 0.168 mmol), 2-Bpinazulene (99.0 mg, 0.390 mmol), Na_2CO_3 (108.0 mg, 1.02 mmol), toluene (5.0 mL), ethanol (5.0 mL), and water (2.0 mL) were placed in the reaction flask and degassed by three freeze-pump-thaw cycles. To the mixture, $Pd(PPh_3)_4$ (21.2 mg, 18.3 μmol) was added under nitrogen flow. The reaction mixture was stirred under reflux condition by heating oil bath (120 °C) for 43 hours. After cooling down to rt, the reaction was diluted with water and toluene. The organic layer was washed with brine, dried over Na_2SO_4, filtered off the desiccant, and concentrated under vacuo. The crude material purified by silicagel column chromatography (eluent hexane : CH_2Cl_2 = 3 : 1). Desired ((2,3-di(azulen-2-yl)-1,4-phenylene)bis(ethyne-2,1-diyl))bis(triisopropylsilane) (**9**, 78.7 mg, 68%) was obtained as a blue solid.

mp > 143 °C (decomp.); ^{1}H NMR (400 MHz, $CDCl_3$) δ 7.97 (d, 4H, *J* = 10.31 Hz), 7.55 (s, 2H), 7.37 (t, 2H, *J* = 9.15 Hz), 7.15 (s, 4H), 6.94 (d, 4H, *J* = 9.15 Hz), 0.84 (s, 42H); ^{13}C NMR (100 MHz, $CDCl_3$): δ 148.81, 140.60, 139.37, 136.11, 135.96, 131.98, 123.82, 122.32, 120.31, 106.53, 95.65, 18.37, 11.11; MS(MALDI-TOF) calcd for $[C_{48}H_{58}Si_2]^+$: 690.41; found: 690.83; Anal. calcd for $C_{48}H_{58}Si_2$: C, 83.41 %; H, 8.46 %; found: C, 83.28 %; H, 8.74 %

2,2'-(3,6-Diethynyl-1,2-phenylene)diazulene: (10)

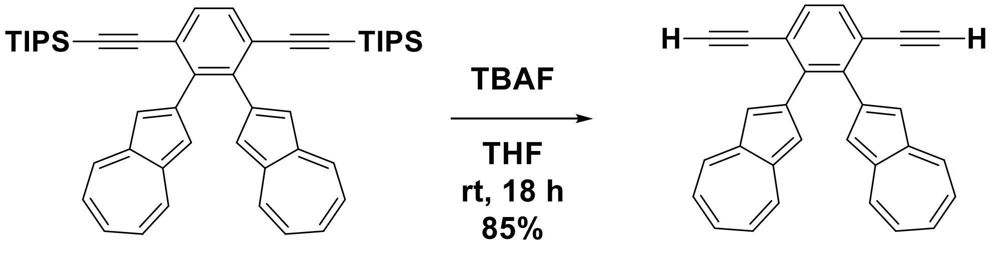

9 10

A 30-mL Schrenk flask was equipped with a magnetic stirring bar, three-way stopcock connected with a vacuum-nitrogen manifold. ((2,3-Di(azulen-2-yl)-1,4-phenylene)bis(ethyne-2,1-diyl))bis(triisopropylsilane) (**9**, 78.7 mg), THF (anhydrous, 2.5 mL), and tetrabutylammonium fluoride (1.0 M THF solution, 0.14 mL, 0.14 mmol) were added to the reaction flask under nitrogen atmosphere. The reaction mixture was stirred for 18 hours at rt. The reaction mixture were diluted with water and CH_2Cl_2, extracted by CH_2Cl_2 (3 times). The combined organic layer was washed with brine, dried over Na_2SO_4, filtered off the desiccant, and concentrated under vacuo. The crude material purified by silicagel column chromatography (eluent hexane : CH_2Cl_2 = 3 : 1). Desired 2,2'-(3,6-diethynyl-1,2-phenylene)diazulene: (**10**, 36.5 mg, 96.4 μmol, 85%) was obtained as a blue solid.

mp > 203 °C (decomp.); ^{1}H NMR (400 MHz, $CDCl_3$) : δ= 8.03 (d, 4H, J = 10.43 Hz), 7.61 (s, 2H), 7.42 (t, 2H, J = 10.43 Hz), 7.13 (s, 4H), 6.98 (t, 4H, J = 9.88 Hz), 3.00 (s, 2H); ^{13}C NMR (100 MHz, $CDCl_3$): δ148.04, 140.82, 139.22, 136.59, 136.37, 132.40, 122.99, 122.65, 120.09, 83.22, 81.65, 18.12, 13.36; MS(EI) calcd for $[C_{30}H_{18}]^+$: 378.14; found: 378

Diazuleno[1,2-*c*:2',1'-*g*]phenanthrene: (1)

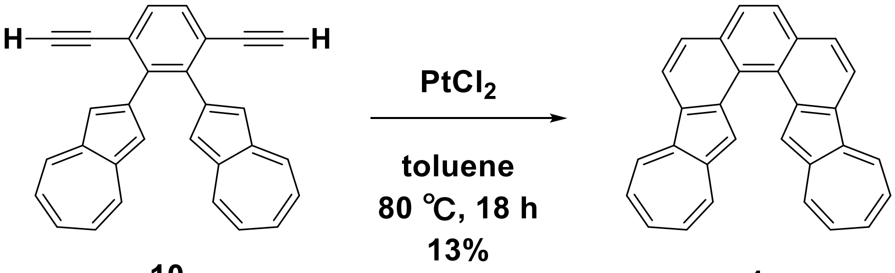

10 1

A 30-mL Schrenk flask was equipped with a magnetic stirring bar, three-way stopcock connected with a vacuum-nitrogen manifold. 2,2'-(3,6-diethynyl-1,2-phenylene)diazulene: (**10**, 36.5 mg, 96.4 μmol), $PtCl_2$ (10.6 mg, 39.9 μmol) were placed in the reaction flask. The reaction flask was flashed with nitrogen, and added toluene (anhydrous and degassed, 3.0 mL). The reaction mixture was stirred for 18 hours at 80 °C. After cooling down to rt, the reaction mixture was

concentrated by evaporation, and subjected to a silicagel column chromatography (eluent hexane : CH_2Cl_2 = 1 : 1). Desired diazuleno[1,2-*c*:2',1'-*g*]phenanthrene: (**1**, 4.7 mg, 12.4 μmol, 13%) was obtained as a green solid.

mp > 152 °C (decomp.); ^{1}H NMR (400 MHz, $CDCl_3$): δ 8.73 (d, 2H, *J* = 8.66 Hz), 8.66 (d, 2H, *J* = 8.60 Hz), 8.34 (s, 2H), 8.19 (d, 2H, *J* = 10.67 Hz), 8.09 (s, 2H), 8.01 (d, 2H, *J* = 8.35 Hz) , 7.45 (t, 2H, *J* = 9.64 Hz), 7.29 (d, 2H, *J* = 10.25 Hz), 7.03 (t, 2H, *J* = 9.39 Hz); ^{13}C NMR (100 MHz, $CDCl_3$): δ 138.83, 137.44, 136.61, 135.57, 133.35, 129.77, 129.56, 127.974, 124.80, 124.42, 123.37, 122.64, 119.90, 119.04; MS(MALDI-TOF) calcd for $[C_{30}H_{18}]^+$: 378.14; found: 378.52; Anal. calcd for $[C_{30}H_{18}+1/6H_2O]$: C, 94.33 %; H, 4.99 %; found: C, 94.48 %; H, 4.97 %.

2,3-Bis(4,4,5,5-tetramethyl-1,3,2-dioxaborolan-2-yl)naphthalene: (12)

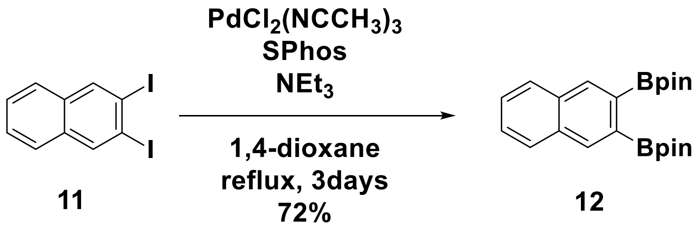

A 30-mL Schrenk flask was equipped with a magnetic stirring bar, three-way stopcock connected with a vacuum-nitrogen manifold. 2,3-Diiodonaphthalene (**11**, 360.8 mg, 0.950 mmol), bis(pinacolato)diboron (0.48 mL, 3.24 mmol), triethylamine (0.9 mL), and 1,4-dioxane (2.0 mL) were placed in the reaction flask and degassed by three freeze-pump-thaw cycles. To the mixture, $PdCl_2(NCCH_3)_2$ (13.0 mg, 50.0 μmol) and SPhos (80.0 mg, 0.20 mmol) were added under nitrogen flow. The reaction mixture was stirred at 110 °C for 3 days. After cooling down to rt, the reaction mixture was diluted with ethyl acetate, filtered with a short plug of cerite, and purified by a silicagel column chromatography (eluent hexane : CH_2Cl_2 = 1 : 1). Desired 2,3-Bis(4,4,5,5-tetramethyl-1,3,2-dioxaborolan-2-yl)naphthalene (**12**, 294.8 mg, 0.776 mmol, 82%) was obtained as a yellow oil.

^{1}H NMR ($CDCl_3$, 25 °C, 400MHz) δ 8.18 (s, 2H), 7.85-7.79(m, 2H), 7.50-7.44 (m, 4H), 1.42 (s, 12H); ^{13}C NMR ($CDCl_3$, 25 °C, 100 MHz) δ 134.47, 133.43, 128.09, 126.53, 83.91, 24.90; HRMS(FAB): calcd. for $[C_{30}H_{18}]^+$; 380.2330 ; Found 380.2333.

Diazuleno[1,2-*a*:2',1'-*c*]anthracene: (2)

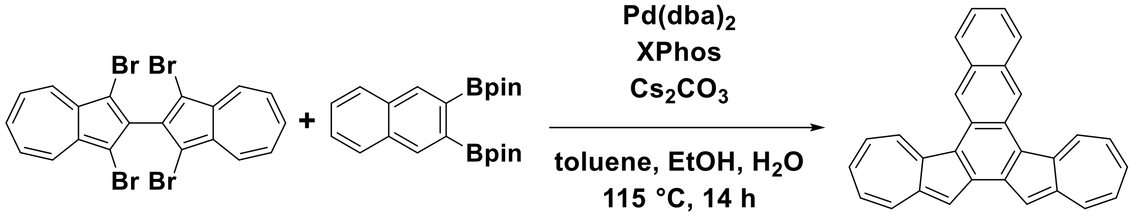

13 **12** **2**

A 30-mL Schrenk flask was equipped with a magnetic stirring bar, three-way stopcock connected with a vacuum-nitrogen manifold. 1,1′,3,3′-Tetraiodo-2,2′-biazulene (**13**, 223.5 mg, 60.0 μmol), 2,3-bis(4,4,5,5-tetramethyl-1,3,2-dioxaborolan-2-yl)naphthalene (**12**, 294.6 mg, 0.094 mmol), Cs_2CO_3 (502.3 mg, 0.25 mmol), toluene (15.0 mL), ethanol (6.0 mL), and water (7.0 mL) were placed in the reaction flask and degassed by three freeze-pump-thaw cycles. To the mixture, $Pd(dba)_2$ (43.7 mg, 12.0 μmol) and XPhos (142.6 mg, 25.0 μmol) were added under nitrogen flow. The reaction mixture was stirred under reflux condition by heating oil bath (120 °C) for 14 hours. After cooling down to rt, the reaction was concentrated under vacuo. The crude mixture was diluted with CH_2Cl_2 and filtered with a short plug of silicagel. The mixture was purified by recycled GPC (JAIGEL 1H-1H, $CHCl_3$). Desired diazuleno[1,2-*a*:2',1'-*c*]anthracene (**2**, 12.2 mg, 32.2 μmol, 14%) was obtained as a brown solid.

mp. 294.7-295.8 °C; ^{1}H NMR ($CDCl_3$, 25 °C, 400 MHz) δ 9.56 (d, J = 9.6 Hz, 2H), 9.41 (s, 2H), 8.55 (d, J = 9.9 Hz, 2H) , 8.18 (m, 2H) , 8.10 (s, 2H) , 7.70 (t, J = 9.7 Hz, 2H) , 7.58 (m, 2H) , 7.51 (t, J = 9.9 Hz, 2H) , 7.31 (t, J = 9.9 Hz, 2H);); ^{13}C NMR ($CDCl_3$, 25 °C, 100 MHz) δ 142.37, 140.62, 137.41, 137.19, 137.15, 135.44, 131.22, 129.22, 127.90, 125.43, 124.93, 124.02, 123.61, 122.31, 113.93.

Calculations

Calculations were conducted using the Materials Studio software suite 2016 (BIOVIA Inc.). The geometries of starting molecules and products were optimized using Dmol^3 module[8, 9] at the B3LYP levels[10–13] of DFT theory with the DNP (ver. 4.4) basis set. Free single molecules were optimized in vacuum without any restriction. Planar molecules (located in x–y plane) were restricted their height z to be zero. Then, the obtained structures were optimized again without any restriction. The formation energies at 298 K and 500 K were calculated at the same level with zero-point energy correction obtained by force calculation.

NICS values of DAPh **1** and DAPy **4** (Supplementary Fig. 5) were calculated using Gaussian 09 rev. E01[14] with GIAO method at B3LYP/6-311G(d,p) level.

Crystallographic Data

Crystallographic data correction of **1**: All measurements were made on a Rigaku R-AXIS RAPID diffractometer using multi-layer mirror monochromated Cu-Kα radiation.

Crystallographic data analysis of **1**: The structures were solved with SIR2004[15] and refined with SHELXL-2013[11]. All calculations were performed by using the Crystal Structure 4.0 crystallographic software package (Rigaku Corporation, Tokyo, Japan). Structural figures of **1** with probability ellipsoids were shown in Supplementary Fig. 16.

Crystallographic data correction of **2**: All measurements were made on a Rigaku Saturn724 diffractometer using multi-layer mirror monochromated Mo-Kα radiation. The crystal-to-detector distance was 44.93 mm.

Crystallographic data analysis of **2**: The structures were solved with SIR2004[15] and refined with SHELXL-2014/3[16]. All calculations were performed by using the Crystal Structure 4.2.2 crystallographic software package (Rigaku Corporation, Tokyo, Japan). Structural figures of **2** with probability ellipsoids were shown in Supplementary Fig. 16.

Supplementary crystallographic information files, which include structure factors, have been deposited with the Cambridge Crystallographic Data Centre (CCDC) as deposition numbers CCDC 1522349, **1**; CCDC 1522350, **2**. These data files can be obtained free of charge from http://www.ccdc.cam.ac.uk/data_request/cif.

Crystal data for **1**: $C_{30}H_{18}$, Formula weight = 378.47, green platelet, 0.25×0.15×0.05 mm^3, monoclinic space group $C2/c$, a = 38.076(2) Å, b = 12.6702(5) Å, c = 7.9552(4) Å, β = 101.488(7)°, V = 3760.9(3) $Å^3$, Z = 8, ρ_{calcd} = 1.337 g·cm^{-3}, μ = 5.765 cm^{-1}, F(000) = 1584, T = 100 K, R_f = 0.0766, wR^2 = 0.1475, 3443 independent reflections [$2\theta \leq 136.4°$] and 343 parameters.

Crystal data for **2**: $C_{30}H_{18}$, Formula weight = 378.47, orange platelet, 0.40×0.20×0.02 mm^3, monoclinic space group $C2/c$, a = 37.63(2) Å, b = 6.361(2) Å, c = 16.724(6) Å, β = 111.123(6)°, V = 3734(3) $Å^3$, Z = 8, ρ_{calcd} = 1.346 g·cm^{-3}, μ = 0.761 cm^{-1}, F(000) = 1584, T = 100 K, R_f = 0.0911, wR^2 = 0.2087, 5377 independent reflections [$2\theta \leq 62.7°$] and 343 parameters.

Supplementary References

1. von Ragué Schleyer, P., Maerker, C., Dransfeld, A., Jiao, H., & van Eikema Hommes, N. J. A Simple and Efficient Aromaticity Probe. *J. Am. Chem. Soc.* **118**, 6317–6318 (1996).
2. Alder, R. W., East, S. P., Harvey, J. N. & Oakley, M. T. The azulene-to-naphthalene rearrangement revisited: A DFT study of intramolecular and radical-promoted mechanisms. *J. Am. Chem. Soc.* **125**, 5375–5387 (2003).
3. Stirling, A., Iannuzzi, M., Laio, A. & Parrinello, M. Azulene-to-naphthalene rearrangement: The car–parrinello metadynamics method explores various reaction mechanisms. *ChemPhysChem* **5**, 1558–1568 (2004).
4. Kislov, V. V. & Mebel, A. M. The Formation of Naphthalene, Azulene, and Fulvalene from Cyclic C_5 Species in Combustion: An Ab Initio/RRKM Study of 9-H-Fulvalenyl (C_5H_5−C_5H_4) Radical Rearrangements. *J. Phys. Chem. A* **111**, 9532–9543 (2007).
5. Diemer, V., Leroux, F. R. & Colobert, F. Efficient and Complementary Methods Offering Access to Synthetically Valuable 1,2-Dibromobenzenes. *Eur. J. Org. Chem.* **2011**, 327–340 (2011).
6. Rodríguez-Lojo, D., Cobas, A., Peña, D., Pérez, D. & Guitián E., *Org. Lett.* **14**, 1363–1365 (2012).
7. Nakae, T., Kikuchi, T., Mori, S., Okujima, T., Murafuji, T. & Uno, H. Bisarylation of 1,1',3,3'-Tetrahalo-2,2'-biazulene under Suzuki–Miyaura Cross-coupling Conditions. *Chem. Lett.* **43**, 504–506 (2014).
8. Delley, B. An all-electron numerical method for solving the local density functional for polyatomic molecules, *J. Chem. Phys.* **92** 508-517 (1990).
9. Delley, B. From molecules to solids with the $DMol^3$ approach, *J. Chem. Phys.* **113**, 7756-7764 (2000).
10. Becke, A. D. Density-functional thermochemistry. III. The role of exact exchange, *J. Chem. Phys.* **98**, 5648-5652, (1993).
11. Lee, C., Yang, W. & Parr, R. G. Development of the Colle-Salvetti correlation-energy formula into a functional of the electron density, *Phys. Rev. B* **37**, 785-789, (1988).
12. Vosko, S. H., Wilk, L. & Nusair, M. Accurate spin-dependent electron liquid correlation energies for local spin density calculations: a critical analysis, *Can. J. Phys.* **58**, 1200-1211, (1980).
13. Stephens, P. J., Devlin, F. J., Chabalowski, C. F. & Frisch, M. J. Ab Initio Calculation of Vibrational Absorption and Circular Dichroism Spectra Using Density Functional Force Fields, *J. Phys. Chem.* **98**, 11623-11627 (1994).
14. Gaussian 09, Revision E.01, M. J. Frisch, G. W. Trucks, H. B. Schlegel, G. E. Scuseria, M. A. Robb, J. R. Cheeseman, G. Scalmani, V. Barone, B. Mennucci, G. A. Petersson, H.

Nakatsuji, M. Caricato, X. Li, H. P. Hratchian, A. F. Izmaylov, J. Bloino, G. Zheng, J. L. Sonnenberg, M. Hada, M. Ehara, K. Toyota, R. Fukuda, J. Hasegawa, M. Ishida, T. Nakajima, Y. Honda, O. Kitao, H. Nakai, T. Vreven, J. A. Montgomery, Jr., J. E. Peralta, F. Ogliaro, M. Bearpark, J. J. Heyd, E. Brothers, K. N. Kudin, V. N. Staroverov, T. Keith, R. Kobayashi, J. Normand, K. Raghavachari, A. Rendell, J. C. Burant, S. S. Iyengar, J. Tomasi, M. Cossi, N. Rega, J. M. Millam, M. Klene, J. E. Knox, J. B. Cross, V. Bakken, C. Adamo, J. Jaramillo, R. Gomperts, R. E. Stratmann, O. Yazyev, A. J. Austin, R. Cammi, C. Pomelli, J. W. Ochterski, R. L. Martin, K. Morokuma, V. G. Zakrzewski, G. A. Voth, P. Salvador, J. J. Dannenberg, S. Dapprich, A. D. Daniels, O. Farkas, J. B. Foresman, J. V. Ortiz, J. Cioslowski, and D. J. Fox, Gaussian, Inc., Wallingford CT, 2013.

15. Burla, M. C., Caliandro, R., Camalli, M., Carrozzini, B., Cascarano, G. L., De Caro, L. Giacovazzo, C., Polidori, G. & Spagna, R. SIR2004: an improved tool for crystal structure determination and refinement. *J. Appl. Cryst.*, **38**, 381–388 (2005).
16. Sheldrick, G. M. A short history of SHELX, *Acta Cryst. A* **64**, 112–122 (2008).
